# ﻿Preface: Proceedings of the 18th International Symposium on Trichoptera

**DOI:** 10.3897/zookeys.1263.177866

**Published:** 2025-12-10

**Authors:** Blanca Ríos-Touma, Paul B. Frandsen, Ralph W. Holzenthal, David C. Houghton, Ernesto Rázuri-Gonzales, Steffen U. Pauls

**Affiliations:** 1 Colegio de Ciencias Biológicas y Ambientales and Global Research & Solutions Center, Universidad San Francisco de Quito, Quito, Ecuador Universidad San Francisco de Quito Quito Ecuador; 2 Brigham Young University, Provo, Utah, USA Brigham Young University Provo United States of America; 3 Department of Entomology, University of Minnesota, Saint Paul, Minnesota, USA University of Minnesota Saint Paul United States of America; 4 Department of Biology, Hillsdale College, Hillsdale, Michigan, USA Hillsdale College Hillsdale United States of America; 5 Senckenberg Research Institute and Natural History Museum Frankfurt, Frankfurt am Main, Germany Senckenberg Research Institute and Natural History Museum Frankfurt Frankfurt am Main Germany; 6 Institute of Insect Biotechnology, Justus-Liebig-University, Gießen, Germany Justus-Liebig-University Gießen Germany

## ﻿Introduction

Since 1974, caddisfly researchers have convened every three years to share developments in all aspects of Trichoptera research, including faunistics, taxonomy, systematics, evolutionary biology, ecology, and biomonitoring. The 18^th^ International Symposium on Trichoptera took place in Quito, Ecuador, from 1 to 5 July 2024. It represented the first caddisfly symposium to be held in South America. Starting with the first meeting of Trichoptera workers initiated by the late Hans Malicky in Lunz am See, Austria, in 1974, the symposium has now been held on all continents except Africa. This special issue constitutes the proceedings from this international conference, highlighting novel insights into the study of the “underwater architects”.

## ﻿The symposium

**Figure 1.**
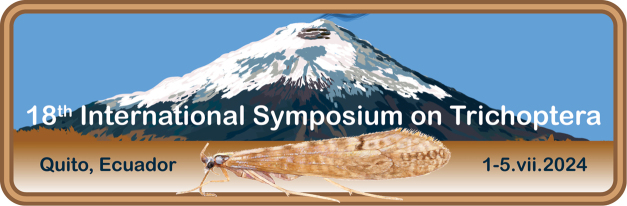
The logo of the 18^th^ International Symposium on Trichoptera, illustrated by Ralph Holzenthal. The logo depicts Volcán Cotopaxi, an iconic feature of the Ecuadorian landscape, and the caddisfly *Nectopsyche paramo*, discovered in this area.

**Figure 2.**
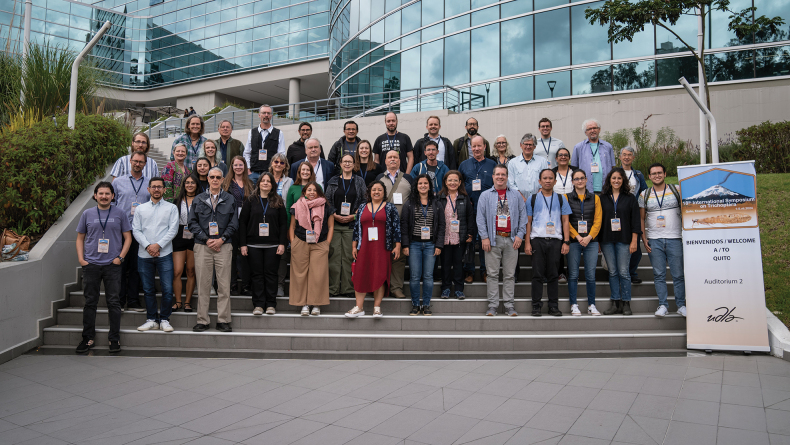
Group photo. Front row, left to right: Mauricio Ramírez-Carmona, Ernesto Rázuri-Gonzales, Naomi Santana, John Morse, Julieta Sganga, Alejandra Correa-Bedoya, Tatiana Latorre-Beltrán, Anne Costa, María Sanabria, Andrew Rasmussen, Pongsak Laudee, Gabriela Jijón, Isabella Errigo, Andrés Castañeda. Second row, left to right: Steffen Pauls, Cyntia Daniela Alvear, Darha Solano-Ulate, Monika Springer, Desiree Robertson, Patina Mendez, Vladimir Ivanov, Albane Vilarino, David Templeman, Ralph Holzenthal, Blanca Ríos-Touma, Mladen Kučinić, Watanabe Shozo. Third row, left to right: Simon Vitecek, Dorothy Bishoff, Megan Bishoff, Gisli Gislason, Robin Thomson. Back row: Debra Finn, François Marie Gibon, David Houghton, Christian Villamarín, Diego Vimos, Fabio Quinteiro, Paul Frandsen, Andrés Morabowen, Jolanda Huisman, Alex Orfinger. Missing: Zahid Hussain, Andrea C. Encalada, Yee Qi Chan, Silvana Gallegos.

The 18^th^ International Symposium on Trichoptera took place at the Universidad de Las Américas (UDLA), located in the Inter-Andean Valley, Quito (Fig. 1). The UDLA Park campus offered a perfect venue for our 18^th^ Symposium, including modern lecture halls, excellent nearby hotels, and easy access from an international airport. Fifty-four participants from 19 countries joined the symposium. The demographic makeup of the meeting was very evenly spaced, with numerous early-career researchers from Latin America attending the Trichoptera Symposium for the first time. At the same time, numerous long-standing regular attendees to previous symposia from North America, Europe, and Asia participated, including Professor John Morse, from Clemson University, who has attended all 18 symposia (Fig. 2).

## ﻿Award recipients

Several institutions provided funding to sponsor the participation of students and young researchers for the Symposium. Applications for sponsorship were numerous, and we had a selection committee composed of Paul Frandsen, Ernesto Rázuri-Gonzales, Christian Villamarín, and Blanca Ríos-Touma. The recipients and the related sponsors were:

Diego Vimos, Universidad de Cuenca (Ecuador), and Cyntia Daniela Alvear, a young student from IKIAM (Universidad Regional Amazónia Ikiam), received support for registration and travel from the World Wildlife Fund. Nature Experience provided three student registrations for Dahra Solano, a student from Universidad de Costa Rica; Silvana Gallegos, from Universidad Nacional de Tucumán, Argentina; and Naomi Santana, from Universidad Tecnológica Indoamérica, Ecuador. Semblis Foundation, Netherlands, provided one registration, granted to Alejandra Correa-Bedoya from Universidad de Antioquia, Colombia.

## ﻿Symposium program

The symposium program was organized by Blanca Ríos-Touma, Ralph W. Holzenthal, Jolanda Huisman, Paul B. Frandsen, Ernesto Rázuri-Gonzales, and Steffen U. Pauls. An opening reception welcomed all participants. The meeting was officially called to order by Blanca Ríos-Touma, the symposium’s organizer and convener. Prof. Gonzalo Mendieta, President of UDLA, gave the welcome remarks and wished the participants a successful meeting. His comments were followed by one of four plenary talks, each starting off the subsequent day’s sessions. These plenary presentations covered central topics in caddisfly research, including freshwater conservation, diversity and distribution, biogeography and faunistics, and systematics and taxonomy:

- Prof. Andrea C. Encalada, Universidad San Francisco de Quito, Ecuador: **Conserving Amazon’s freshwater health, biodiversity, and connectivity**
- Prof. Debra Finn, Missouri State University, United States: **Stream networks, dispersal traits, and connectivity in mountain stream headwaters**
- Prof. Monika Springer, Universidad de Costa Rica, Costa Rica: **Trichoptera studies: perspectives from the Central American land bridge**
- Prof. Simon Vitecek, University of Natural Resources and Life Sciences, Austria: **The importance of taxonomy and systematics (again) and some future opportunities for Trichoptera research**

Each day included morning and afternoon sessions of contributed papers and a session devoted to posters. The contributed oral and poster presentations covered the fields of taxonomy, systematics, and evolution (15), diversity and faunistics (17), and ecology and biomonitoring (17). The symposium program, including abstracts of all presentations, has been digitally archived at https://doi.org/10.5281/zenodo.17665618. Also, a calming video of caddisflies in flight was prepared by Rogier Maaskant of Rotterdam, The Netherlands, and was displayed before each day’s presentations. A formal dinner was held in the old colonial part of Quito at the “Purísima” Restaurant on the last day of scientific presentations (Fig. 8). The symposium ended on 5 July with participants choosing between two one-day excursions to the Cotopaxi National Park or the Tandayapa Biological Reserve. This allowed participants to enjoy and marvel at Ecuador’s natural beauty and remarkable biodiversity (Figs 10, 11).

List of contributed oral presentations in alphabetical order of the first author (presenter in bold face; * denotes presentations published in the proceedings)

- **Yee Qi Chan**, Yuchen Ang, Bryna J.Y. Liang, Darren C.J. Yeo & John C. Morse: Trichoptera in Singapore: A first look
- **Alejandra Correa-Bedoya** & Fernando J. Muñoz-Quesada: Temporal and spatial comparative analysis of taxonomic richness and functional diversity of Trichoptera in urban and rural waters of middle and lower Cauca Basin, Colombia
- Everton S. Dias, Adolfo R. Calor, **Albane Vilarino** & Pitagoras C. Bispo: Diversification and historical biogeography of long-horned caddisflies (Trichoptera: Leptoceridae)
- **Isabella M. Errigo**, Andrés Morabowen, Paul B. Frandsen & Blanca Rios-Touma: eDNA in the Neotropics: Testing its efficacy as a biomonitoring tool
- **Paul B. Frandsen**: What do genes have to do with caddisfly silk?
- **Paul B. Frandsen** & **Ralph W. Holzenthal**: Milne and Milne revisited: The evolution of caddisflies*
- Sonja Gerwin, Xiling Deng, Fengzhi He & **Steffen U. Pauls**: Elevational diversity of *Rhyacophila* (Trichoptera: Rhyacophilidae) in the Hengduan Shan*
- **Gísli Már Gíslason** & Snaebjörn Pálsson: Relationship of Trichoptera species in Iceland with other North-Atlantic islands and the mainland of Europe*
- **Ralph W. Holzenthal** & Blanca Ríos-Touma: The Trichoptera of Ecuador
- **David C. Houghton**: Analyzing adult Trichoptera to assess upstream disturbance: one (caddis) metric to rule them all?*
- **Zahid Hussain**, Aquib Majeed, Tabraq Ali, Sajad H. Parey & Manpreet S. Pandher: Taxonomy and DNA barcodes of the family Philopotamidae (Trichoptera: Insecta) from India
- **Vladimir Ivanov**, Stanislav Melnitsky & Andrey A. Przhiboro: First data on Trichoptera of the Putorana Plateau (Northern Siberia)
- **Gabriela Jijón**, Isabella M. Errigo, Jessica Wicks, Natalie Nyborg, Lillian Buck, Daniel Davis, Sam Standring, John Chaston, Blanca Rios-Touma & Paul B. Frandsen: Morphology outperforms DNA barcoding in identifying Trichoptera in Ecuadorian streams
- Mathias Kümmerlen & **Steffen U. Pauls**: Higher predicted climate-change vulnerability for spring-dwelling freshwater biota*
- **Tatiana Latorre Beltrán**, William Gerth, Ivan Arismendi & Blanca Ríos-Touma: Trichoptera dispersion: insights from lateral and longitudinal sampling
- **Pongsak Laudee** & Pimpajee Kaewwong: Species diversity of caddisflies (Insecta: Trichoptera) from lowland forest springs, Surat Thani Province, southern Thailand
- **Patina K. Mendez**: Growth and development of undergraduate Trichoptera researchers*
- **Andrés Morabowen**, Blanca Ríos-Touma, Isabella M. Errigo & Paul B. Frandsen: Land-use and elevation-driven changes in caddisfly assemblages in Neotropical streams in Ecuador
- **Alexander Orfinger**, Truc Bui & Andrew Rasmussen: Larval taxonomy of the net-spinning caddisfly *Cernotina truncona* Ross, 1947 (Trichoptera: Polycentropodidae)
- **Fabio B. Quinteiro**, Adolfo R. Calor, Gleison R. Desidério, Leandro L. Dumas, Ana Lucia Henriques-Oliveira, Rafael Pereira, Ana Maria Pes, Allan P.M. Santos & Albane Vilarino: Exploring the Trichoptera in the Taxonomic Catalog of the Brazilian Fauna: recent progress and perspectives
- **Mauricio Ramírez-Carmona**, Atilano Contreras-Ramos & Robin E. Thomson: Microcaddisflies (Trichoptera: Hydroptilidae) of the Baja California peninsula, Mexico, and their biogeographic affinities*
- **Andrew K. Rasmussen**, Dana R. Denson, Alexander B. Orfinger & Steven C. Harris: The Trichoptera fauna of Florida: diversity and distribution
- **Ernesto Rázuri-Gonzales**, Roger J. Blahnik, François Ngera Mwangi & Steffen U. Pauls: The Trichoptera of Africa: New species, unknowns, and state-of-the-art
- **Blanca Ríos-Touma**, Ralph W. Holzenthal, Paul Frandsen, Steffen U. Pauls: Elevational biodiversity gradients in caddisflies from the tropical Andes
- **Desiree Robertson-Thompson**: Caddisflies in hot water: a review of climate change studies related to Trichoptera
- **Darha Solano-Ulate** & Monika Springer: Waterfalls as a reservoir for caddisfly larvae: exploring a poorly known habitat*
- **David Tempelman** & María J. Sanabria: Trichoptera and Citizen Science in the Netherlands*

List of contributed poster presentations in alphabetical order of the first author (presenter in bold face; * denotes presentations published in the proceedings)

- **Cyntia Daniela Alvear Sayavedra**, Mariana Vellosa Capparelli & Rodrigo Espinosa: Aquatic macroinvertebrate diversity and water quality along an anthropic gradient in the upper Napo River Basin
- **Andrés Castañeda**, Christian Villamarín & Blanca Ríos-Touma: Trichopteran community changes across different land uses and diets of *Mortoniella* sp. (Trichoptera: Glossosomatidae) & *Smicridea* sp. (Trichoptera: Hydropsychidae) in Andean rivers
- Anne M. Costa, Laisse Moura, Leandro Juen & **Fábio B. Quinteiro**: Filling the Linnean and Wallacean gaps in Eastern Amazon: two new species of *Polyplectropus* Ulmer, 1905 (Trichoptera: Polycentropodidae) and new distributional records of other polycentropodids
- Pedro López Del Castillo, Perla Alonso Eguía-Lis, Liliana María Gómez Luna & Germán M. López Iborra (presented by **Monika Springer**): Microhabitat use and seasonality of caddisflies (Trichoptera: Insecta) in two streams in eastern Cuba
- **Silvana Gallegos-Sánchez** & Iván Jácome-Negrete: Assessing trichopteran diversity in the Oglán River watershed: Insights from Amazonian Kichwa communities
- Xinyu Ge & **John C. Morse**: Functional traits of the ancestral caddisfly larva*
- William Gerth, Christina Murphy, Ivan Arismendi & **Tatiana Latorre-Beltrán**: Using submerged light traps to learn about caddisflies that dive to oviposit around the world
- **Mladen Kučinić**: Contribution to the fauna, distribution, and DNA barcoding data of caddisflies (Insecta: Trichoptera) in Croatia*
- Stella Li, Colette Christensen & **Patina K. Mendez**: Caddisfly species identification via wing morphometrics
- Aquib Majeed, Tabraq Ali, **Zahid Hussain**, Sajad H. Parey & Manpreet S. Pandher: DNA barcoding and taxonomic insights of subfamily Hydropsychinae (Hydropsychidae: Trichoptera: Insecta) from India
- **Rogier Maaskant**: “Sense of Presence” (special photographic presentation)
- **Mauricio Ramírez-Carmona** & Robin E. Thomson: Towards a robust phylogeny of Stactobiinae (Trichoptera: Hydroptilidae): synthesizing molecular and morphological data
- **Ernesto Rázuri-Gonzales**: The caddisflies of the Great African Lakes: An example of Lakes Albert and Malawi
- **Ernesto Rázuri-Gonzales** & Ralph W. Holzenthal: New *Atopsyche* (Hydrobiosidae) from Peru*
- **Naomi Santana**, Carla Díaz & Rebeca Paredes: Assessment of macroinvertebrate communities and water quality in Northwest Quito Metropolitan District
- Jan Simon Stark, Oskar Schröder & **Steffen U. Pauls**: Integrating genetic diversity in temporal insect monitoring: The example of three freshwater invertebrate species in the Bavarian Forest National Park*
- **Julieta V. Sganga**, Cecilia Brand, Juan J. Morrone & Danielle Anjos-Santos: On the Andean endemic genus *Scotiotrichia* Mosely (Trichoptera: Glossosomatidae: Protoptilinae): description of the larva and biogeographic patterns of the Andean Protoptilinae
- **Julieta V. Sganga**, Gleison R. Desidério, Camila Fornis, María Laura Libonatti, Fabián G. Jara, Gabrielle Jorge & Neusa Hamada: Exploring the diversity of caddisflies (Insecta: Trichoptera) in Patagonia: new records and taxonomic insights from Argentina
- **Robin E. Thomson**: Microcaddisflies, morphology, and modern molecular methods: collections-based research to establish the microcaddisfly phylogeny
- **Christian Villamarín**, Agnes Lohs, Mishell Donoso, Blanca Ríos-Touma, Pablo Castillejo, Melanie Loachamin & Milton Sosa: Sublethal effects of two heavy metals on *Nectopsyche* sp. (Trichoptera: Leptoceridae) in Andean rivers
- **Diego Vimos**, Pablo V. Mosquera, Henrietta Hampel & Raúl F. Vazquez: Spatial distribution of Trichoptera larvae in the Cajas Massif lakes
- **Shozo Watanabe**: Estimate of the life history and the net spinning habit on *Macrostemum radiatum* (McLachlan 1872) in the central Honshu Island of Japan

### ﻿List of symposium participants

- Cyntia Daniela Alvear, Universidad Regional Amazónica Ikiam, Ecuador
- Dorothy Bishoff, Clemson University, U.S.A.
- Megan Bishoff, Clemson University, U.S.A.
- Andrés Castañeda, Universidad de Las Américas, Ecuador
- Yee Qi Chan, National University of Singapore, Singapore
- Alejandra Correa-Bedoya, Universidad de Antioquia, Colombia
- Anne Costa, Universidade Federal do Pará, Brazil
- Andrea C. Encalada, Universidad San Francisco de Quito, Ecuador
- Isabella Errigo, Cornell University, U.S.A.
- Debra Finn, Missouri State University, U.S.A.
- Paul B. Frandsen, Brigham Young University, U.S.A.
- Silvana Gallegos-Sánchez, Ecoforensic CIC, Ecuador
- François-Marie Gibon, Institute de Recherche pour le Développement, France
- Gísli Már Gíslason, University of Iceland, Iceland
- Ralph W. Holzenthal, University of Minnesota, U.S.A.
- David C. Houghton, Hillsdale College, U.S.A.
- Jolanda Huisman, University of Minnesota, U.S.A.
- Zahid Hussain, Baba Ghulam Shah Badshah University, India
- Vladimir Ivanov, Saint Petersburg State University, Russia
- Gabriela Jijón, Brigham Young University, U.S.A.
- Mladen Kučinić, University of Zagreb, Croatia
- Tatiana Latorre-Beltrán, Oregon State University, U.S.A.
- Pongsak Laudee, Prince of Songkla University, Thailand
- Patina Mendez, University of California Berkeley, U.S.A.
- Andrés Morabowen, Universidad de Las Américas; Universidad San Francisco de Quito, Ecuador
- John Morse, Clemson University, U.S.A.
- Alex Orfinger, Dalton State College, U.S.A.
- Steffen U. Pauls, Senckenberg Research Institute and Natural History Museum Frankfurt, Germany
- Fabio Quinteiro, Universidade Federal do Pará, Brazil
- Mauricio Ramírez-Carmona, University of Minnesota, U.S.A.
- Andrew Rasmussen, Florida A&M University, U.S.A.
- Ernesto Rázuri-Gonzales, Senckenberg Research Institute and Natural History Museum Frankfurt, Germany
- Blanca Ríos-Touma, Universidad de Las Américas; Universidad San Francisco de Quito, Ecuador
- Desiree Robertson, U.S. Geological Survey, U.S.A.
- Maria Sanabria, Stichting Semblis, The Netherlands
- Naomi Santana, Universidad Tecnológica Indoamérica, Ecuador
- Julieta Sganga, Universidad de Buenos Aires, Argentina
- Watanabe Shozo, Independent, Japan
- Darha Solano-Ulate, Universidad de Costa Rica, Costa Rica
- Monika Springer, Universidad de Costa Rica, Costa Rica
- David Templeman, Stichting Semblis, The Netherlands
- Robin Thomson, University of Minnesota, U.S.A.
- Albane Vilarino, Universidade de São Paulo, Brazil
- Christian Villamarín, Universidad de Las Américas, Ecuador
- Diego Vimos, Universidad de Cuenca, Ecuador
- Simon Vitecek, University for Natural Resources and Life Sciences Vienna, Austria

## ﻿In memoriam

In the time between the 17^th^ International Symposium on Trichoptera in Lunz am See, Austria (5–9 September 2022) and the 18^th^ Symposium in Quito (1–5 July 2024) we lost several colleagues who will be greatly missed.

Fernando Muñoz-Quesada (1956–2024) was Professor at the University of Antioquia in Medellín, Colombia. A native of San José, Costa Rica, Fernando graduated from the University of Costa Rica where he worked in the ichthyology collection and at Costa Rica’s National Biodiversity Institute (INBio) on a project to survey the caddisflies of the country. He later attended the University of Minnesota where he revised the New World species of *Wormaldia*. He also described new species in several other genera from Costa Rica, including *Leptonema*, *Austrotinodes*, and *Xiphocentron*). After receiving his PhD in 2002, he spent the rest of his career at the University of Antioquia teaching and mentoring students in aquatic entomology. He was a caring and attentive teacher, loved by his students. He was known for his humor and his love of Latin American literature and culture. An obituary is planned by his students and colleagues at the University of Antioquia.

Carmen Zamora Muñoz (1964–2022) was a Professor of Zoology at the University of Granada in Spain. Born in Sevilla, she attended the same university at which she later spent her academic career. Her contributions to the taxonomy, biogeography, and ecology of Trichoptera of the Iberian Peninsula provided fundamental knowledge of evolutionary ecology and aquatic biodiversity of the region. Her 30-year career spanned research in Europe, North Africa, and the Americas. In addition to her dedication to teaching at the University of Granada, Carmen was recognized for her rigor, passion, and commitment to science, leaving an important legacy. An obituary by Núria Bonada, Javier Alba-Tercedor, and Marcos González is available in Limnetica (https://www.limnetica.com/documentos/limnetica/limnetica-42-1-i.pdf) as is one by Marcos González in Braueria (https://www.zobodat.at/pdf/BRA_50_0005-0010.pdf).

Dave Ruiter (1948–2021) was an expert on western North American caddisflies, with works that expanded to taxa distributed across the globe, including Japan. While Dave was perhaps best known for his work on Limnephilidae, including his 1995 treatment of the taxonomy of new world *Limnephilus*, his work encompassed many other groups of caddisflies. He had a particular interest in associating females to species and did so for many North American species. Dave is remembered by his colleagues as an enthusiastic researcher who was generous with his time in helping others. An obituary for Dave was written by his wife, Terri, and colleagues and is available in the Society for Freshwater Science news (https://freshwater-science.org/news/in-memorium-david-ernest-ruiter).

Sára Nógrádi (1942–2023) was an expert on the caddisfly fauna of Hungary and the Carpathian Basin. Her research focused on the faunistics of the region which culminated in 75 scientific publications and the fundamental book for the region “The Caddisflies of Hungary (Trichoptera)” which she wrote together with her husband Ákos Uherkovich. She was known for her detailed illustrations, many of which were published in the Caddisflies of Hungary. An obituary was written in Braueria in 2024 by Ákos Uherkovich (https://www.zobodat.at/pdf/BRA_51_0051-0052.pdf).

Hans Malicky (1935–2025) was an Emeritus Associate Professor of the University of Vienna and retired scientist of the Biological Station Lunz of the Austrian Academy of Sciences. Born in Theresienfeld and trained in Vienna, Hans was likely the most prolific caddisfly taxonomist of the last few decades, perhaps ever. With > 2500 new species described mainly from Europe, Asia, and Africa, he was well-known to the entire caddisfly community. He initiated the First International Symposium on Trichoptera, of which the Trichoptera Newsletter Braueria was an outcome. For 52 issues it served as both a publication outlet for faunistic and taxonomic studies, but also as an important repository for literature on caddisflies as well as information on caddisfly workers around the globe. While likely best known for his taxonomic work and his Atlas of European Trichoptera, Hans was also an excellent freshwater ecologist and biogeographer. He was also very generous in sharing his time and knowledge with colleagues from across the globe, many of whom visited him in his home in Lunz am See. An extensive obituary is currently in preparation.

David Allen (“Ets”) Etnier (1938–2023) was Professor Emeritus from the University of Tennessee, USA. He joined the faculty after completing his PhD at the University of Minnesota on the caddisflies of the state. He is best known for his discovery of the snail darter (*Percina tanasi*), a previously unknown species of fish in the path of a proposed federal dam project, thus testing the limits of the U.S. Endangered Species Act. Ets was an energetic instructor, researcher, and naturalist. His Regional Studies classes provided several generations of Tennessee biologists with active opportunities to learn about biogeography, bird songs, freshwater mussel taxonomy, and aquatic insect ecology, among other topics. His Trichoptera research included faunistic studies on the caddisflies of Minnesota and several southeastern US states as well as taxonomic studies on the genera *Hydropsyche*, *Pycnopsyche*, *Agapetus*, *Neophylax*, *Rhyacophila*, and several other genera. Prior to his death, he and his wife Elizabeth endowed the University of Tennessee with perpetual support for his fish and caddisfly collection, housed in the Department of Ecology and Evolutionary Biology.

Charles Rex (“Chuck”) Parker (1948–2023) was a biologist in Great Smoky Mountains National Park, USA. After his dissertation work at Virginia Polytechnical, where he examined the ecology of filter feeding caddisflies in some Virginia rivers, Chuck pursued postdoctoral work at the Royal Ontario Museum in Toronto. His employment in the park lasted 30 years under the umbrella of several different federal agencies. Chuck was a patient and keenly observant naturalist who added immense value to every project. He was instrumental in the historic All Taxa Biotic Inventory in the Great Smokies and partnered with specialists in many different insect taxa to leverage the ATBI effort to generate great scientific discoveries. He revised the world species of the odontocerid genus *Psilotreta*, the limnephilid genus *Hesperophylax*, the goerid genus *Goerita*, and the glossosomatid genus *Agapetus* in eastern N. America.

**Figure 3.**
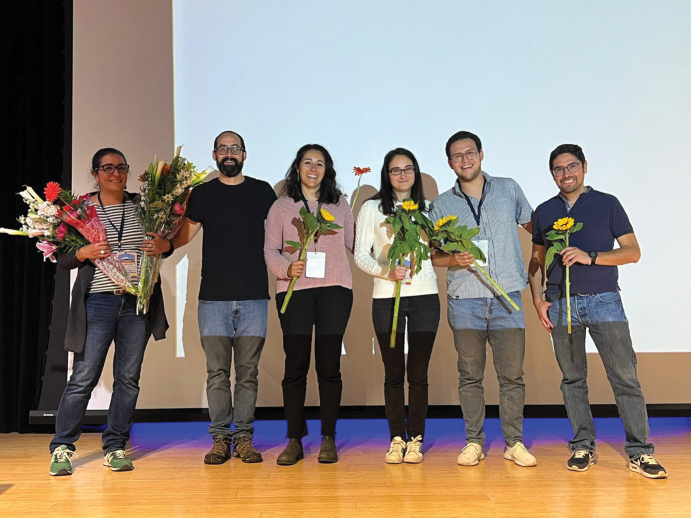
Local arrangements committee. Blanca Ríos-Touma, Andrés Morabowen, Isabella Errigo, Gabriela Jijón, Andrés Casteñeda, Christian Villamarín.

**Figure 4.**
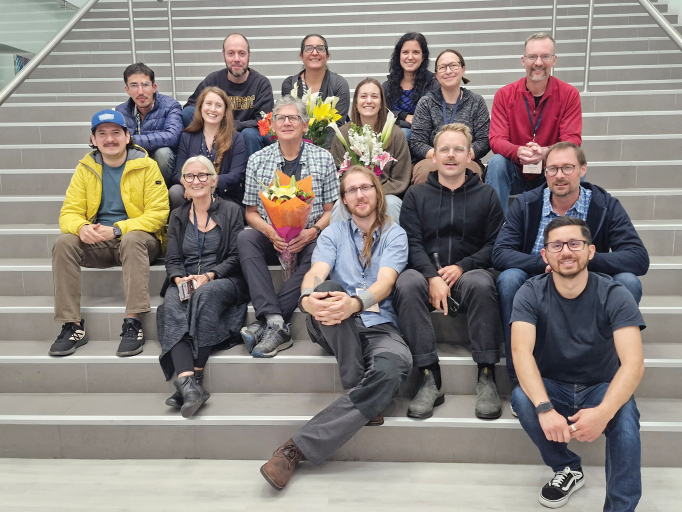
Graduates and associates of the Holzenthal lab. Left to right. Mauricio Ramírez (Mexico), Albane Vilarino (Brazil), Jolanda Huisman (USA, Netherlands), Desiree Robertson-Thompson (USA), Fabio Quinteiro (Brazil), Ralph Holzenthal (USA), Blanca Ríos-Touma (Ecuador), Simon Vitecek (Austria), Robin Thomson (USA), Anne Costa (Brazil), Paul Frandsen (USA), Patina Mendez (USA), Ernesto Rázuri-Gonzales (Peru, Germany), Steffen Pauls (Germany), David Houghton (USA).

**Figure 5.**
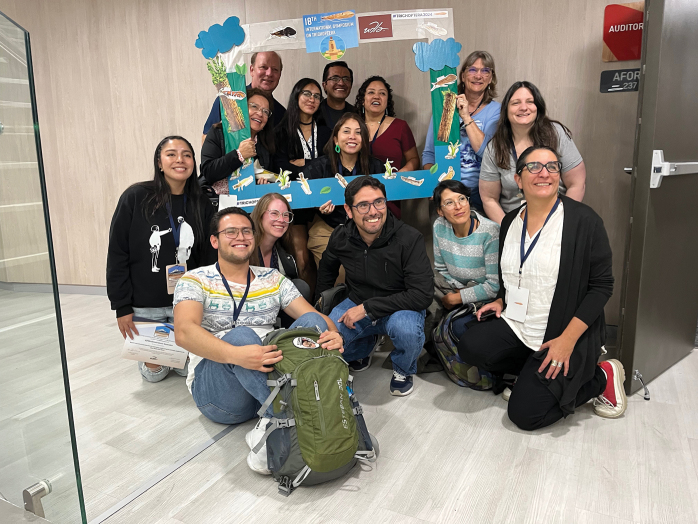
Latin American participants. Upper row: María Judith Sanabria (Colombia, The Netherlands), David Tempelman (The Netherlands), Naomi Santana (Ecuador), Alejandra Correa-Bedoya (Colombia), Diego Vimos (Ecuador), Tatiana Latorre (Colombia, USA), Monika Springer (Costa Rica), Julieta Sganga (Argentina). Lower row: Cyntia Daniela Alvear (Ecuador), Andrés Castañeda (Ecuador), Dahra Solano (Costa Rica), Christian Villamarín (Ecuador), Silvana Gallegos (Ecuador), Blanca Ríos-Touma (Ecuador).

**Figure 6.**
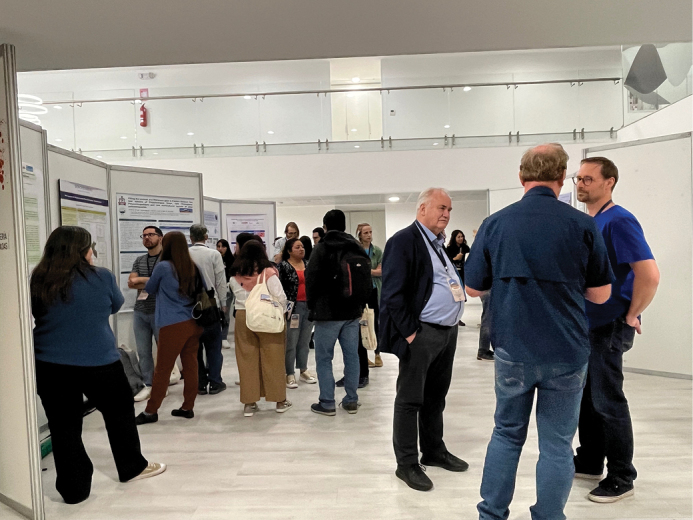
Poster session. Foreground, Gísli Gíslason (Iceland), David Templeman (Netherlands), Steffen Pauls (Germany).

**Figure 7.**
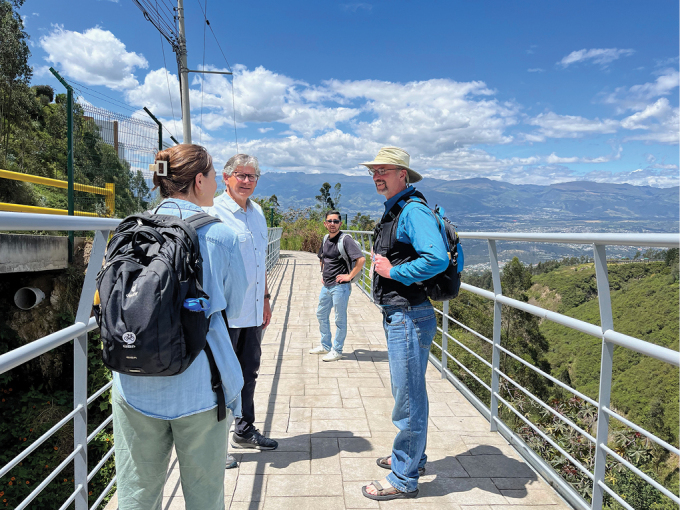
UDLA Ecotrail. Robin Thomson (USA), Ralph Holzenthal (USA), Ernesto Rázuri-Gonzales (Peru, Germany), David Houghton (USA).

**Figure 8.**
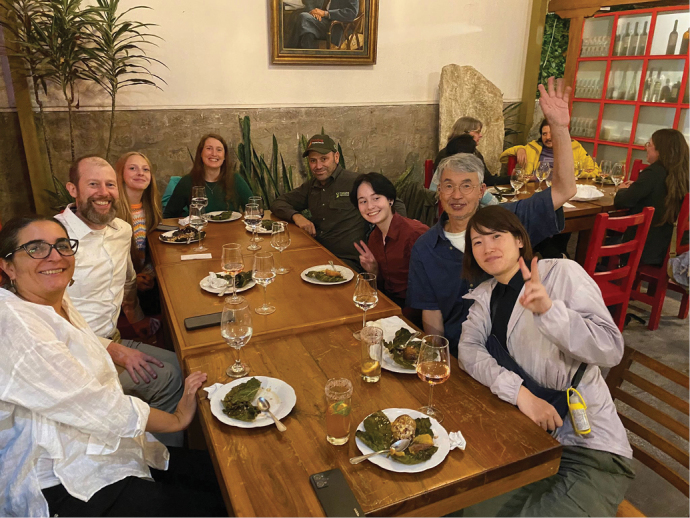
Symposium dinner, left to right. Blanca Ríos-Touma (Ecuador), Alex Thompson (USA), Lucinda Thompson (USA), Desiree Robertson-Thompsom (USA), Xavier Amigo (Ecuador), Stella-Clara Thompson (USA), Watanabe Shozo (Japan), Watanabe Mizuki (Japan).

**Figure 9.**
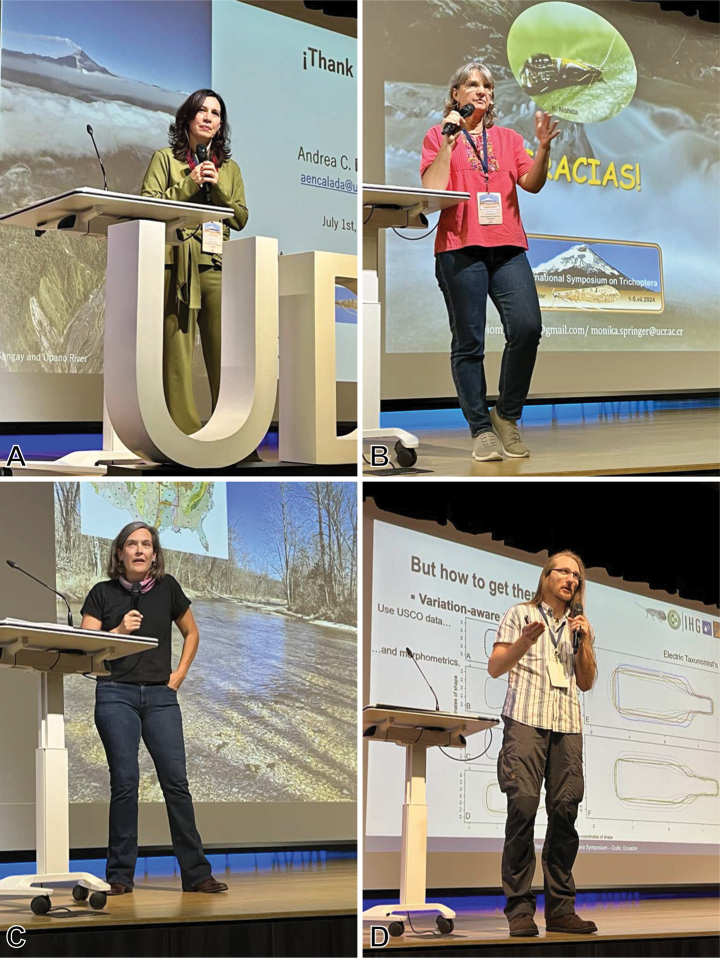
Plenary speakers. A. Andrea Encalada (Ecuador); B. Monika Springer (Costa Rica); C. Debra Finn (USA); D. Simon Vitecek (Austria).

**Figure 10.**
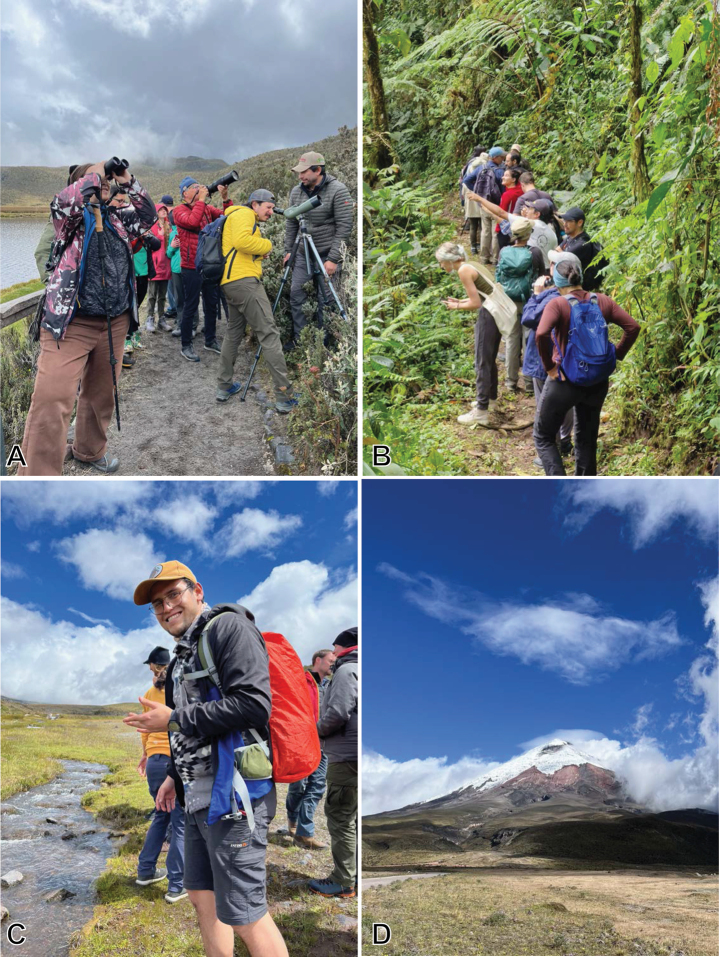
Field trips. A. Bird watching in the páramo, Cotopaxi National Park; B. Hiking in the cloud forest, Bellavista Reserve; C. Andrés Casteñeda, Cotopaxi National Park; D. Cotopaxi Volcano.

**Figure 11.**
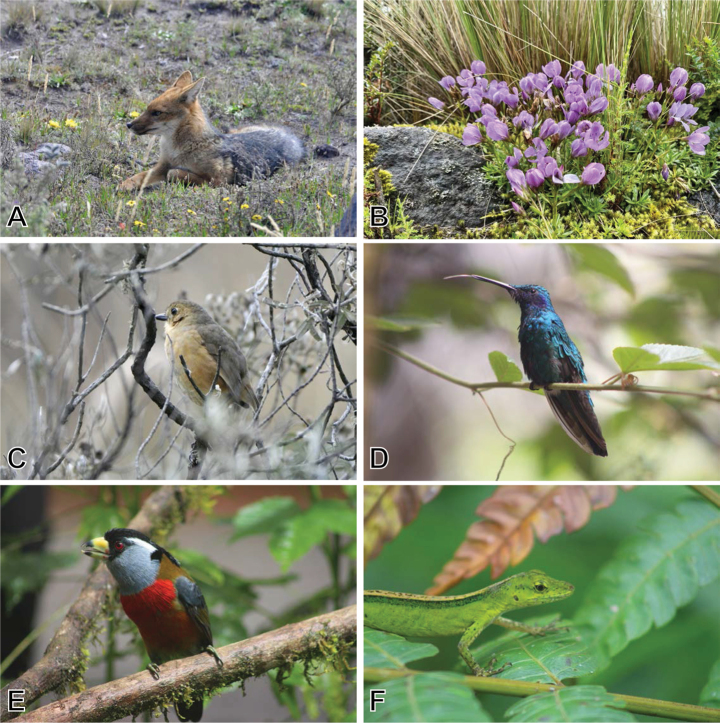
Ecuadorian biodiversity, Cotopaxi National Park, and Alambi cloud forest. A. Andean fox, *Lycalopex culpaeus*; B. Dwarf gentian, *Gentianella cerastioides*; C. Tawny Antpitta, *Grallaria quitensis*; D. Sparkling Violetear, *Colibri coruscans*; E. Toucan barbet, *Semnornis ramphastinus*; F. Gem anole, *Anolis gemmosus*.

## ﻿The proceedings

As with the conference, the ZooKeys Special Issue covers topics from evolution and phylogenetics to ecology, faunistics, and taxonomy of Trichoptera. In the individual papers, two themes are recurring: first, it is clear that we are still missing substantial basic knowledge about the diversity and distribution of caddisflies around the globe. This is true for adult and immature life stages, and even in regions with a long history of aquatic entomology research, e.g. Europe and North America. In the proceedings, five new species are described from North America. However, as can be expected, 22 new species are described from hyperdiverse and less intensively studied regions in Latin America. Evidently, we are still scratching at the surface of species and ecological diversity in caddisflies. Second, increasing the interdisciplinarity of the research being conducted is opening new avenues. For example, faunistics and taxonomy reveal many new species (even in putatively well-studied regions), are often conducted hand-in-hand, and increasingly focus on integrative methodologies.

The scope of the proceedings is wide-ranging with two, five, eight, and five contributions on the Trichoptera of Asia, Europe, Latin America, and North America, respectively. Two contributions address Trichoptera as a whole.

### ﻿Taxonomy, systematics, and evolution

Both Ge and Morse (2025) and Frandsen and Holzenthal (2025) look at the deep and early evolution of Trichoptera using very recent phylogenetic insights to better understand the more than 250 million years of caddisfly evolution and ecological diversification.

No less than eight studies in these proceedings exemplify the lack of basic knowledge of species-level caddisfly diversity, particularly regarding the aquatic stages. New species descriptions are combined with faunistic distribution data and new records for Brazil (Quinteiro et al. 2025), Peru (Rázuri-Gonzales and Holzenthal 2025), Ecuador (Holzenthal et al. 2025; Thomson et al. 2025), and Mexico (Ramírez-Carmona et al. 2025). Moreover, while the North American Trichoptera are relatively well-studied (e.g. Hogan et al. 2025), Rasmussen et al. (2025) show that a county-level synopsis of caddisflies from the state of Florida reveal numerous new species and four new state records. Solano-Ulate and Springer (2025) provide a first assessment of Neotropical caddisflies associated with waterfalls, showcasing the diversity of this previously understudied extreme environment. The diversity of *Rhyacophila* larvae in the Hengduan Shan mountains (China) can be assessed only at the genus level or using species surrogates, such as molecular operational taxonomic units (Gerwin et al. 2025).

### ﻿Diversity and faunistics

Chan et al. (2025) assess the diversity of caddisflies in the streams of Singapore across a landcover gradient, providing important faunistic information of this freshwater-poor island state. Kučinić et al. (2025) provide an overview of the Trichoptera Fauna of Croatia, integrating faunistic, biogeographic, and DNA barcoding data to bring the total number of known species to 225, thereby tripling the number of recorded species in the past three decades. Similarly, Hogan et al. (2025) show that much is known about the diversity and distributions of caddisflies in the United States of America and Canada, allowing a future focus on underrepresented areas, species complexes likely to harbor cryptic diversity, or larval taxonomy to improve freshwater biomonitoring.

With shorter, more streamlined tertiary education, which also covers ever-increasing numbers of topics, learning about organismal diversity is often less than in past decades. At the same time, it is difficult for university researchers to build long-lasting research programs with short-term undergraduate students. Houghton (2025) and Mendez (2025) outline ideas on how this can be achieved and provide impressive scientific outputs that come from building consistent data sets over longer periods of time. Tempelman et al. (2025) show that long-term data can alternatively be generated through broad public engagement. Harnessing the power of citizen science has the potential to educate and empower interested entomology amateurs, while providing valuable monitoring data, even for comparatively poorly known insect groups like caddisflies.

### ﻿Ecology and biomonitoring

Habitat preferences and how colonization and dispersal processes occur are at the center of López López Del Castillo et al. (2025) and Latorre-Beltrán et al. (2025) and central for understanding present-day distributions. These patterns are also a product of historical dispersal events and environmental change (Gíslason and Pálsson 2025). While knowledge remains limited for many regions and species, spatial inferences already provide initial insights into how climate change may influence future distribution patterns (Kuemmerlen et al. 2025).

Finally, Trichoptera are being used to help monitor change at levels of organization from genes (Stark et al. 2025), to microbiomes (Jijón et al. 2025), to Trichoptera species, genera, and families (Houghton 2025), and even to assess the impact of climate change based on data collected by citizen scientists (Tempelman et al. 2025).

From the symposium and these proceedings, it is clear that we are unlikely to run out of new caddisfly species discoveries, novel aquatic insect biodiversity assessments, or ecological and evolutionary questions that can and should be addressed with Trichoptera for a long time to come.

## ﻿Additional information

### Use of AI

No use of AI was reported.

### Author ORCIDs

Blanca Ríos-Touma https://orcid.org/0000-0002-3921-0908

Paul B. Frandsen https://orcid.org/0000-0002-4801-7579

Ralph W. Holzenthal https://orcid.org/0000-0003-1853-6340

David C. Houghton https://orcid.org/0000-0001-6946-4864

Ernesto Rázuri-Gonzales https://orcid.org/0000-0002-7554-0816

Steffen U. Pauls https://orcid.org/0000-0002-6451-3425

## Supplementary materials

Supplementary material 1 Conference program Data type pdf https://binary.pensoft.net/file/1487343This dataset is made available under the Open Database License (http://opendatacommons.org/licenses/odbl/1.0/). The Open Database License (ODbL) is a license agreement intended to allow users to freely share, modify, and use this Dataset while maintaining this same freedom for others, provided that the original source and author(s) are credited.Blanca Ríos-Touma, Paul B. Frandsen, Ralph W. Holzenthal, David C. Houghton, Ernesto Rázuri-Gonzales, Steffen U. Pauls

## References

[B1] Chan YQ Liang BJY Ang Y Morse JC Cai Y Yeo DCJ (2025) Diversity, abundance and distribution of caddisfly (Insecta, Trichoptera) families in relation to environmental parameters across freshwater streams in Singapore. In: Ríos-Touma B Frandsen PB Holzenthal RW Houghton DC Rázuri-Gonzales E Pauls SU (Eds) Proceedings of the 18th International Symposium on Trichoptera.ZooKeys 1263: 21–36. 10.3897/zookeys.1263.147968 PMC12712629 41426544

[B2] López Del Castillo P López Iborra GM Gómez Luna LM Alonso Eguía-Lis P (2025) Micro-habitat use and seasonality of caddisfly larvae (Trichoptera) in two streams in eastern Cuba. In: Ríos-Touma B Frandsen PB Holzenthal RW Houghton DC Rázuri-Gonzales E Pauls SU (Eds) Proceedings of the 18th International Symposium on Trichoptera.ZooKeys 1263: 333–350. 10.3897/zookeys.1263.150346 PMC12712631 41426543

[B3] Ge X Morse JC (2025) Functional traits of ancestral caddisfly (Trichoptera) larvae and pupae. In: Ríos-Touma B Frandsen PB Holzenthal RW Houghton DC Rázuri-Gonzales E Pauls SU (Eds) Proceedings of the 18th International Symposium on Trichoptera.ZooKeys 1263: 47–68. 10.3897/zookeys.1263.148069 PMC12712640 41426527

[B4] Gerwin S Deng X He F Pauls SU (2025) Diversity of *Rhyacophila* (Trichoptera, Rhyacophilidae) in the Hengduan Mountains. In: Ríos-Touma B Frandsen PB Holzenthal RW Houghton DC Rázuri-Gonzales E Pauls SU (Eds) Proceedings of the 18th International Symposium on Trichoptera.ZooKeys 1263: 69–88. 10.3897/zookeys.1263.153111 PMC12712619 41426548

[B5] Gíslason GM Pálsson S (2025) Relationship of Trichoptera species in Iceland with Europe and North America. In: Ríos-Touma B, Frandsen PB, Holzenthal RW, Houghton DC, Rázuri-Gonzales E, Pauls SU (Eds) Proceedings of the 18th International Symposium on Trichoptera. ZooKeys 1263: 89–104. 10.3897/zookeys.1263.148150 PMC12712624 41426542

[B6] Hogan PN Houghton DC Murray-Stoker K Dewalt RE Rasmussen AK Morse JC (2025) An analytical synopsis of caddisfly (Insecta, Trichoptera) taxonomic history and progress in Canada and the United States. In: Ríos-Touma B, Frandsen PB, Holzenthal RW, Houghton DC, Rázuri-Gonzales E, Pauls SU (Eds) Proceedings of the 18th International Symposium on Trichoptera. ZooKeys 1263: 105–122. 10.3897/zookeys.1263.147986 PMC12712632 41426534

[B7] Holzenthal RW Blanhik RJ Ríos-Touma B (2025) New Philopotamidae (Insecta, Trichoptera) from Ecuador: seven new species and updated country checklist. In: Ríos-Touma B Frandsen PB Holzenthal RW Houghton DC Rázuri-Gonzales E Pauls SU (Eds) Proceedings of the 18th International Symposium on Trichoptera.ZooKeys 1263: 123–145. 10.3897/zookeys.1263.147996 PMC12712617 41426538

[B8] Houghton DC (2025) Predicting landscape disturbance using adult Trichoptera: one (caddis) metric to rule them all? In: Ríos-Touma B Frandsen PB Holzenthal RW Houghton DC Rázuri-Gonzales E Pauls SU (Eds) Proceedings of the 18th International Symposium on Trichoptera.ZooKeys 1263: 147–164. 10.3897/zookeys.1263.141377 PMC12712643 41426530

[B9] Jijón G Hough C Gedris D Frandsen PB Chaston JM (2025) Gut microbiome composition of Trichoptera larvae across functional feeding groups: a case study from the Provo River, Utah, USA. In: Ríos-Touma B Frandsen PB Holzenthal RW Houghton DC Rázuri-Gonzales E Pauls SU (Eds) Proceedings of the 18th International Symposium on Trichoptera.ZooKeys 1263: 165–177. 10.3897/zookeys.1263.147980 PMC12712634 41426531

[B10] Kučinić M Previšić A Ćukušić A Vučković I Žalac S Cerjanec D Ćuk R Stojanovic KZ Akimbekova N Vuković M Skejo J Hlebec D Božić A Kutnjak H (2025) Fauna, distribution, and DNA barcoding data of caddisflies (Insecta, Trichoptera) in Croatia. In: Ríos-Touma B Frandsen PB Holzenthal RW Houghton DC Rázuri-Gonzales E Pauls SU (Eds) Proceedings of the 18th International Symposium on Trichoptera.ZooKeys 1263: 179–288. 10.3897/zookeys.1263.152515 PMC12712641 41426536

[B11] Kuemmerlen M Graf W Waringer J Vitecek S Kučinić M Previšić A Keresztes L Bálint M Pauls SU (2025) Higher predicted climate-change vulnerability for spring-dwelling freshwater biota. In: Ríos-Touma B Frandsen PB Holzenthal RW Houghton DC Rázuri-Gonzales E Pauls SU (Eds) Proceedings of the 18th International Symposium on Trichoptera.ZooKeys 1263: 289–315. 10.3897/zookeys.1263.148253 PMC12712645 41426533

[B12] Latorre-Beltrán T Arismendi I Rios-Touma B Gerth WJ Petty A (2025) Lateral and longitudinal dispersal of aquatic insects in mountain streams, with notes about Trichoptera. In: Ríos-Touma B Frandsen PB Holzenthal RW Houghton DC Rázuri-Gonzales E Pauls SU (Eds) Proceedings of the 18th International Symposium on Trichoptera.ZooKeys 1263: 317–331. 10.3897/zookeys.1263.150229 PMC12712621 41426546

[B13] Mendez PK (2025) Workflow designs can facilitate sample management for student-research teams doing taxonomic resource development with Trichoptera. In: Ríos-Touma B Frandsen PB Holzenthal RW Houghton DC Rázuri-Gonzales E Pauls SU (Eds) Proceedings of the 18th International Symposium on Trichoptera.ZooKeys 1263: 351–364. 10.3897/zookeys.1263.150420 PMC12712620 41426540

[B14] Quinteiro FB Costa AM Saldanha G Moura L Assunção OT Juen L (2025) A new species of *Oecetis* McLachlan, 1877 (Trichoptera, Leptoceridae) and new distributional records of Trichoptera in the eastern Amazon. In: Ríos-Touma B Frandsen PB Holzenthal RW Houghton DC Rázuri-Gonzales E Pauls SU (Eds) Proceedings of the 18th International Symposium on Trichoptera.ZooKeys 1263: 365–377. 10.3897/zookeys.1263.151613 PMC12712616 41426526

[B15] Rasmussen AK Denson DR Orfinger AB Harris SC (2025) Diversity and distribution of the Trichoptera of Florida, United States, with descriptions of five new species. In: Ríos-Touma B Frandsen PB Holzenthal RW Houghton DC Rázuri-Gonzales E Pauls SU (Eds) Proceedings of the 18th International Symposium on Trichoptera.ZooKeys 1263: 389–439. 10.3897/zookeys.1263.147317 PMC12712626 41426545

[B16] Rázuri-Gonzales E Holzenthal RW (2025) The genus *Atopsyche* (Trichoptera, Hydrobiosidae) in Peru, with the description of seven new species. In: Ríos-Touma B Frandsen PB Holzenthal RW Houghton DC Rázuri-Gonzales E Pauls SU (Eds) Proceedings of the 18th International Symposium on Trichoptera.ZooKeys 1263: 441–478. 10.3897/zookeys.1263.150396 PMC12712636 41426535

[B17] Solano-Ulate D Springer M (2025) Waterfalls as a reservoir for caddisfly larvae (Insecta, Trichoptera): exploring a poorly known habitat. In: Ríos-Touma B Frandsen PB Holzenthal RW Houghton DC Rázuri-Gonzales E Pauls SU (Eds) Proceedings of the 18th International Symposium on Trichoptera.ZooKeys 1263: 479–498. 10.3897/zookeys.1263.148087 PMC12712644 41426532

[B18] Stark JS Schröder O Müller J Seifert L Pauls SU (2025) Temporal monitoring of genetic diversity in aquatic insects: a pilot study in the Bavarian Forest National Park. In: Ríos-Touma B Frandsen PB Holzenthal RW Houghton DC Rázuri-Gonzales E Pauls SU (Eds) Proceedings of the 18th International Symposium on Trichoptera.ZooKeys 1263: 499–518. 10.3897/zookeys.1263.147797 PMC12712642 41426547

[B19] Tempelman D Verberk WCEP Sanabria MJ (2025) Citizen science reveals a shift in the commonness and rarity of Trichoptera in the Netherlands. In: Ríos-Touma B Frandsen PB Holzenthal RW Houghton DC Rázuri-Gonzales E Pauls SU (Eds) Proceedings of the 18th International Symposium on Trichoptera.ZooKeys 1263: 519–530. 10.3897/zookeys.1263.147805 PMC12712637 41426529

[B20] Thomson RE Ríos-Touma B Holzenthal RW (2025) Additions to the genus *Rhyacopsyche* Müller, 1879 (Trichoptera, Hydroptilidae) in Ecuador. In: Ríos-Touma B Frandsen PB Holzenthal RW Houghton DC Rázuri-Gonzales E Pauls SU (Eds) Proceedings of the 18th International Symposium on Trichoptera.ZooKeys 1263: 531–543. 10.3897/zookeys.1263.148084 PMC12712635 41426539

