# Supplementary material for: ﻿Preface: Proceedings of the 18th International Symposium on Trichoptera
Source: Zookeys. 2025 Dec 10;1263:1–19. doi: 10.3897/zookeys.1263.177866 (PMC12712624; doi:10.3897/zookeys.1263.177866)
Supplement: Supplementary material 1 — Conference program [file zookeys-1263-001_article-177866__-s001.pdf]

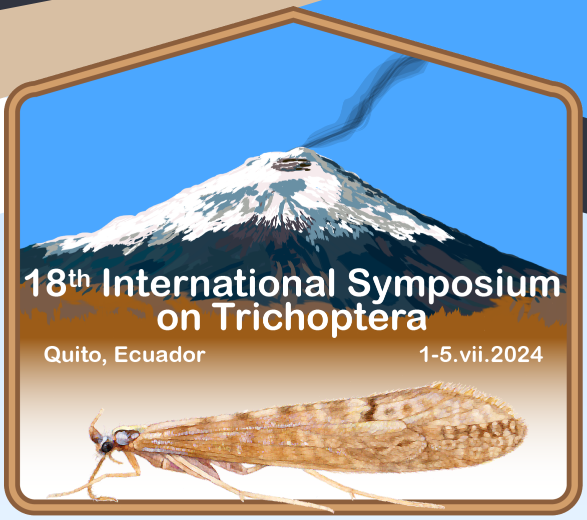

PROGRAM & ABSTRACTS

18th International Symposium on Trichoptera

Universidad de Las Américas
Quito, Ecuador
1-5 July 2024

	Monday July 1st	Tuesday July 2nd	Wednesday July 3rd	Thursday July 4th	Friday July 5th
8:00-9:00	Registration	Registration	Registration	Registration	Excursion
Moderator		Ralph Holzenthal	Steffen Pauls	Paul Frandsen	
9:00-9:20	Registration	Monika Springer	Simon Vitecek	Deb Finn	
9:20-9:40	opening sessions	Monika Springer	Simon Vitecek	Deb Finn	
9:40-10:00	opening sessions	Monika Springer	Simon Vitecek	Deb Finn	
10:00-10:30	Break	Break	Break	Break	
**Moderator**	Blanca Ríos-Touma	Andrés Morabowen	Isabella Errigo	Tatiana Latorre	
10:30-10:50	Andrea Encalada	Ramírez	Gerwin (Pauls)	Orfinger	
10:50-11:10	Andrea Encalada	Dias (Vilarino)	Gislason	Chan (remote)	
11:10-11:30	Andrea Encalada	Quiteiro	Houghton	Kümmerlen (Pauls)	
11:30-11:50	Holzenthal	Frandsen 1	Latorre-Beltrán	Robertson	
			**Group Photography**		
12:00-12:30	Lunch Break	Lunch Break	Lunch Break	Lunch Break	
12:30-12:50	Lunch Break	Lunch Break	Lunch Break	Lunch Break	
12:50-13:10	Lunch Break	Lunch Break	Lunch Break	Lunch Break	
13:10-13:30	Lunch Break	Lunch Break	Lunch Break	Lunch Break	
13:30-13:50	Lunch Break	Lunch Break	Lunch Break	Lunch Break	
13:50-14:00	Lunch Break	Lunch Break	Lunch Break	Lunch Break	
**Moderator**	Ernesto Rázuri-Gonzál	David Tempelman	Fabio Quinteiro	Steffen Pauls	
14:00-14:30	Errigo	Ríos-Touma	Rázuri	Hussain	
14:30-14:50	Jijon	Mendez	Morabowen	Clossing Ceremony	
14:50-15:10	Correa-Bedoya	Rasmussen	Frandsen 2	Clossing Ceremony	
15:10-15:40	Break	Break	Break	Break	
Moderator	Mauricio Ramírez				
15:40-16:10	Laudee	Poster Session	Poster Session		
16:10-16:30	Ivanov	Poster Session	Poster Session		
16:30-16:50	Solano-Ulate	Poster Session	Poster Session		
16:50-17:10	Tempelman	Poster Session	Poster Session		
17:00-17:20		Poster Session	Poster Session		
17:20-17:40					
17:40-18:00	Welcome Reception				
18:00-19:00					
19:00-20:00			Symposium Dinner		
20:00 onwards					

SCHEDULE
18th International Symposium on Trichoptera

Monday, July 1st

Opening Session

8:00-9:20	Registration
9:20-10:00	Welcome addresses: Dr. Blanca Ríos-Touma, Docente Investigador and Convenor, Universidad de Las Américas, Dr. Gonzalo Mendieta, Rector, Universidad de Las Américas; announcements and general information.
10:00-10:30	Break

Plenary Address

Moderator, Blanca Ríos Touma

10:30-11:30	Dr. Andrea Encalada, Universidad San Francisco de Quito, Ecuador. *Conserving Amazon's Freshwater Health, Biodiversity and Connectivity*

Presentations

11:30-11:50	Ralph Holzenthal, *The Trichoptera of Ecuador*
12:00-14:00	Lunch

Moderator, Ernesto Rázuri-Gonzales

14:00-14:30	Isabella Errigo, *eDNA in the Neotropics: Testing its efficacy as a biomonitoring tool*
14:30-14:50	Gabriella Jijón, *Morphology outperforms DNA barcoding in identifying Trichoptera in Ecuadorian streams*
14:50-15:10	Alejandra Correa-Bedoya, *Temporal and spatial comparative analysis of taxonomic richness and functional diversity of Trichoptera in urban and rural waters of middle and lower Cauca Basin, Colombia*
15:10-15:40	Break

Moderator, Mauricio Ramírez

15:40-16:10	Pongsak Laudee, *Species diversity of caddisflies (Insecta: Trichoptera) from lowland forest springs, Surat Thani Province, southern Thailand*
16:10-16:30	Vladimir Ivanov, *First data on Trichoptera of the Putorana Plateau (Northern Siberia)*
16:30-16:50	Darha Solano-Ulate, *Waterfalls as a reservoir for caddisfly larvae: exploring a poorly known habitat*
16:50-17:10	David Templeman, *Trichoptera and Citizen Science in the Netherlands*

Welcome Reception

Universidad de Las Américas
17:40

Tuesday, July 2nd

8:00-9:00 Registration

Plenary Address

Moderator, Ralph Holzenthal

9:00-10:00 Prof. Monika Springer, Universidad de Costa Rica. *Trichoptera studies: Perspectives from the Central American land bridge*

10:00-10:30 Break

Presentations

Moderator, Andrés Morabowen

10:30-10:50 Mauricio Ramírez, *Towards a robust phylogeny of Stactobiinae (Trichoptera: Hydroptilidae): synthesizing molecular and morphological data*
10:50-11:10 Everton Dias (Albane Vilarino), *Diversification and historical biogeography of long-horned caddisflies (Trichoptera: Leptoceridae)*
11:10-11:30 Fabio Quinteiro, *Exploring the Trichoptera in the Taxonomic Catalog of the Brazilian Fauna: recent progress and perspectives*
11:30-11:50 Paul Frandsen, *What do genes have to do with caddisfly silk?*

12:00-14:00 Lunch

Moderator, David Templeman

14:00-14:30 Blanca Ríos-Touma, *Elevational biodiversity gradients in caddisflies from the tropical Andes*
14:30-14:50 Patina Mendez, *Growth and development of undergraduate Trichoptera researchers*
14:50-15:10 Andrew Rasmussen, *The Trichoptera fauna of Florida: diversity and distribution*

15:10-15:40 Break

15:40-17:20 **Poster Session 1** [Ali, Alvear, Costa, Del Castillo, Ge, Gerth, Kučinić, Maaskant, Rázuri-Gonzales, Santana, Sganga, Villamarín]

Wednesday, July 3rd

8:00-9:00 Registration

Plenary Address

Moderator, Steffen Pauls

9:00-10:00 Dr. Simon Vitecek, University of Natural Resources and Life Sciences, Austria. *The importance of taxonomy and systematics (again) and some future opportunities for Trichoptera research*

10:00-10:30 Break

Presentations

Moderator, Isabella Errigo

10:30-10:50 Sonja Gerwin (Pauls), *Elevational diversity of Rhyacophila (Trichoptera: Rhyacophilidae) in the Hengduan Shan*

10:50-11:10 Gísli Már Gíslason, *Relationship of Trichoptera species in Iceland with other North-Atlantic islands and the mainland of Europe*

11:10-11:30 David Houghton, *Analyzing adult Trichoptera to assess upstream disturbance: one (caddis) metric to rule them all?*

11:30-11:50 Tatiana Latorre-Beltrán, *Trichoptera dispersion: insights from lateral and longitudinal sampling*

12:00-14:00 Lunch

Moderator, Fabio Quinteiro

14:00-14:30 Ernesto Rázuri-Gonzales, *The Trichoptera of Africa: New species, unknowns, and state-of-the-art*

14:30-14:50 Andrés Morabowen, *Landuse and elevation-driven changes in caddisfly assemblages in Neotropical streams in Ecuador*

14:50-15:10 Paul Frandsen, *Milne and Milne revisited: The evolution of caddisflies*

15:10-15:40 Break

15:40-17:20 **Poster Session 2** [Castañeda, Gallegos, Li, Majeed, Maaskant, Ramírez, Rázuri, Sganga, Stark, Thomson, Vimos, Watanabe]

Symposium Dinner
La Purísima, Quito Historic District

19:00

Thursday, July 4th

8:00-9:00 Registration

Plenary Address

Moderator, Paul Frandsen

9:00-10:00 Dr. Deb Finn, Missouri State University, USA. *Stream networks, dispersal traits, and connectivity in mountain stream headwaters*

10:00-10:30 Break

Presentations

Moderator, Tatiana Latorre-Beltrán

10:30-10:50 Alexander Orfinger, *Larval taxonomy of the net-spinning caddisfly Cernotina truncona Ross, 1947 (Trichoptera: Polycentropodidae)*

10:50-11:10 Yee Qi Chan, *Trichoptera in Singapore: A first look*

11:10-11:30 Mathias Kümmerlan (Steffen Pauls), *Higher predicted climate-change vulnerability for spring-dwelling freshwater biota*

11:30-11:50 Desiree Robertson, *Caddisflies in hot water: a review of climate change studies related to Trichoptera*

12:00-14:00 Lunch

Moderator, Steffen Pauls

14:00-14:30 Zahid Hussain, *Taxonomy and DNA barcodes of the family Philopotamidae (Trichoptera: Insecta) from India*

14:30-15:10 Closing Session: Discussion of Proceedings, venue for 19th Symposium, information about excursions, announcements and other items; closing remarks

Friday, July 5th

Excursions

Tandayapa Valley and Volcán Cotopaxi

PLENARY SPEAKERS

Dr. Andrea C. Encalada
Universidad San Francisco de Quito, Ecuador

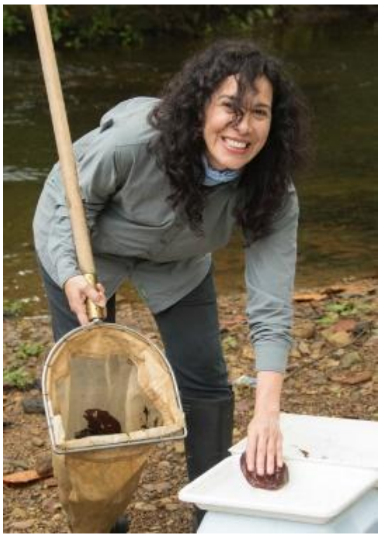

Andrea C. Encalada is a freshwater ecologist and professor at Universidad San Francisco de Quito and an Adjunct Professor at the University of North Carolina Chapel Hill. She got her Ph.D. from Cornell University, and since then, she has been working on stream and river research in tropical and temperate ecosystems. During the last 15 years, she has focused her research on river structure and function along elevation gradients in Andean-Amazon watersheds. She is particularly interested in climate change and other anthropogenic changes, including drying river networks, and how these impacts might alter populations, communities, and ecosystem processes.

Conserving Amazon's Freshwater Health, Biodiversity and Connectivity*

The Amazon Basin, a cornerstone of global biodiversity and a critical regulator of ecological processes, is facing unprecedented threats from human activities and climate change. Amazonian freshwater ecosystems provide invaluable services, including water purification, transportation, energy, and food production, along with carbon sequestration and sustaining an immense biodiversity. The basin's hydrological cycles, driven by 'aerial rivers,' recycle a significant portion of water and contribute to continental rainfall patterns.

These ecosystems host an incredible array of biodiversity; approximately 2,500 fish species inhabit these waters, many of which are endemic and vital to the livelihoods of local communities. In spite of this, many aquatic groups remain understudied.

Despite their importance, Amazonian freshwater ecosystems are rapidly degrading due to pollution, deforestation, dam construction, mining, and climate change. The lack of sewage treatment facilities, substandard environmental practices in mining and oil extraction, and the construction of hydroelectric dams are fragmenting rivers, reducing biodiversity, and impairing ecosystem functions. These activities not only affect aquatic life but also have severe socio-economic impacts on Indigenous Peoples and Local Communities (IPLCs).

To address these challenges, we advocate for the conservation, remediation, and restoration of Amazonian freshwater ecosystems. Key recommendations include halting dam construction and promoting sustainable energy by implementing a moratorium on new dam projects and investing in decentralized sustainable energy solutions that support local economies and preserve ecological functions. Enhancing water treatment and pollution control is essential, with urgent development of water treatment infrastructure, enforcement of pollution control policies, and promotion of the restoration of riparian vegetation to improve water quality and ecosystem health.

Integrating climate change strategies into regional planning is crucial to maintaining ecosystem resilience and connectivity. Empowering IPLCs by recognizing and integrating Indigenous knowledge with scientific approaches enhances conservation efforts and promotes co-management of freshwater resources. Establishing transnational governance agreements and securing international financial support are vital for cohesive management and conservation efforts across the Amazon Basin. Investing in science and innovation supports cross-disciplinary research and technological innovations to better understand and mitigate stressors on freshwater ecosystems.

By fostering collaborative efforts among scientists, policymakers, IPLCs, and stakeholders, we aim to develop robust strategies to protect and restore the health and connectivity of Amazonian freshwater ecosystems. This integrative approach is crucial for sustaining the ecological balance and ensuring the well-being of both the environment and local communities. Here, I will explore these vital topics and discuss how we can collectively contribute to a sustainable future for the Amazon Basin's freshwater ecosystems.

*also presented on behalf of the Science Panel for the Amazon (SPA).

Dr. Deb Finn
Missouri State University, United States

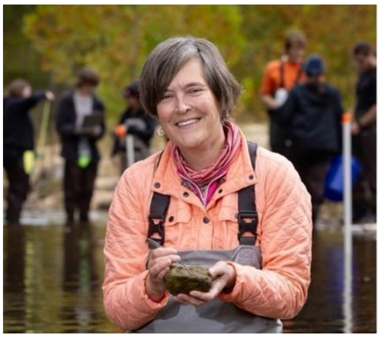

Deb is a stream ecologist and associate professor at Missouri State University (USA). She spends most of her time attempting to mesh empirical research in streams with teaching and mentoring students, ultimately aiming to fill them with awe over "stream bugs". She is fascinated by the phenomenal diversity of stream invertebrates that persists in such dynamic physical habitats, and much of her work addresses the roles of connectivity and habitat heterogeneity in conferring resistance and resilience to populations. Mountain headwaters have long been her focus, given the complex topography, hydrological heterogeneity, and physical isolation of headwaters at the tips of stream networks. She has worked in the tropical high Andes of Ecuador, the Alps and Pyrenees of Europe, the Great Dividing Range in SE Australia, and a number of mountainous regions of North America. Her favorite taxa are aquatic insects, particularly the EPT.

Stream networks, dispersal traits, and connectivity in mountain stream headwaters

Mountain streams are fascinating and informative systems for testing hypotheses about spatial distributions of organisms. Steep upstream-downstream environmental gradients, combined with water sources that can have strongly contrasting physicochemical characteristics, produce high levels of diversity over short spatial extents. High-elevation headwater branches of stream networks can also be isolated from one another by harsh terrestrial conditions at drainage divides, further promoting beta diversity.

In this talk, I'll start with a brief overview of my background and findings associated with aquatic insect diversity in mountain streams, and I'll place strong emphasis on the role of dispersal limitation in generating spatial patterns of diversity in aquatic insects (definitely including caddisflies!) within and among stream networks. I'll then present some case studies from both temperate and tropical high-mountain systems, and associated with both intraspecific diversity at the population-genetic level and interspecific patterns of community diversity. These case studies will suggest a few take-home messages, including: 1) At the single-species level, knowing something about dispersal/movement capacity and degree of habitat specialization provides much insight into how genetic diversity is distributed spatially. 2) It is probably unwise to infer dispersal from "proxy" traits such as insect wing morphometrics. Although more difficult to measure, the only way to understand dispersal is to study elements of dispersal itself. 3) At the community level, aquatic habitat heterogeneity and complex landscape topography produce an intricate mosaic of environmental conditions in mountain streams, including some conditions that are expected to be substantially more resilient to global change than others. We will then converge on a general conclusion that habitat heterogeneity and connectivity allowing organism movement among habitat patches is and will continue to be essential for maintaining diversity in stream-dwelling insects through broad-scale environmental shifts associated with climate change.

Prof. Monika Springer
Universidad de Costa Rica, Costa Rica

Monika is a biologist who graduated from the University of Munich, Germany. She arrived in Costa Rica for the first time in 1990 as an exchange student, and since 1995 she has worked at the School of Biology of the University of Costa Rica, where she is a full professor. She is also an associate researcher at the Center for Research in Marine Sciences and Limnology, CIMAR, and curator of Aquatic Entomology in the Museum of Zoology, University of Costa Rica (MZUCR) located in the Center for Research in Biodiversity and Tropical Ecology (CIBET). Her research interests include the taxonomy and biology of aquatic insects, emphasizing caddisfly larvae and their use as biological indicators in monitoring, managing, and conserving freshwater environments.

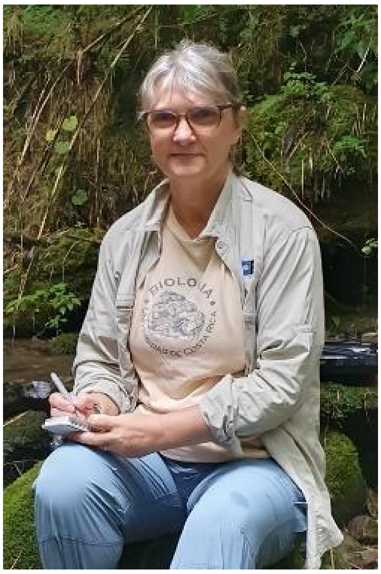

Trichoptera studies: Perspectives from the Central American land bridge

The Central American land bridge, which extends from southern Mexico to the northeast of Colombia, is part of the Mesoamerican region and includes the countries of Guatemala, Belize, Honduras, El Salvador, Nicaragua, Costa Rica, and Panama (from north to south). Due to its geological history, position between two oceans, and variable topography, this region hosts many terrestrial and aquatic ecosystems and is considered one of the world's Biodiversity hotspots. As several authors pointed out, Central America is considered a hyper-diverse area within the Neotropical region, harboring 5-12% of the world's species richness. However, when comparing scientific production on biodiversity topics, there are significant differences between the northern countries and their southern neighbors, Costa Rica and Panama, which produce almost five times as many peer-reviewed scientific papers. This pattern also applies to our knowledge of caddisflies, with less information published from El Salvador, Belize, Honduras, and Nicaragua. A literature review was conducted based on the *Catalog of the Neotropical Trichoptera*, published by Holzenthal and Calor in 2017, and subsequent publications to compile specific information for each country. To date, 15 families and 61 genera have been recorded from the seven Central American countries, with a total of almost 800 species, 545 representing new species. As a result of a former 10-year inventory by Holzenthal and co-workers in Costa Rica, the country accounts for 295 holotypes, while 179 belong to Panama, reflecting the recent and ongoing efforts made by Armitage and collaborators. For the past nine years, this research team has doubled the amount of Trichoptera species for Panama and added 2 families and 11 genera to its inventory, reaching now a total of 533 species from 56 genera. This makes Panama the country with the most caddisfly species within the region, followed closely by Costa Rica, which hosts the highest number of species per area, with nine species per 1000 km^2. The remaining five countries show significantly lower numbers, and, together with the fact that 30% of the species were collected so far in only one country, this is most certainly an indicator of the low sampling effort in most Central American countries, as stated also by several other authors. As for the immature stages, knowledge is even less, with only 7% of all Central American species having their larval stage associated and described, despite their well-known importance as biological indicators in rivers and streams. On the other hand, due to their widespread use in biomonitoring programs and environmental impact studies, a vast amount of information is being produced on the abundance and distribution of caddisfly larvae in various water bodies. Collaborative research efforts, capacity building, and the commitment to hosting local, well-maintained collections are necessary to improve our knowledge of the highly threatened Central American freshwater biodiversity, identify conservation gaps, and help achieve more integrative watershed management in this especially vulnerable region.

Dr. Simon Vitecek
University of Natural Resources and Life Sciences, Austria

Simon is an aquatic entomologist and mostly concentrates on caddisflies (but can easily be distracted by stoneflies, mayflies and other aquatic invertebrates). After starting out on a purely taxonomic and systematic research agenda as part of a fantastic team, a lot of his work now deals with aquatic insect biodiversity in general: Simon tries to understand how ecological and evolutionary processes shape biodiversity patterns but also how biodiversity shapes ecosystem functioning. But sometimes he doesn't and spends hours in marvel at the beauty of aquatic life.

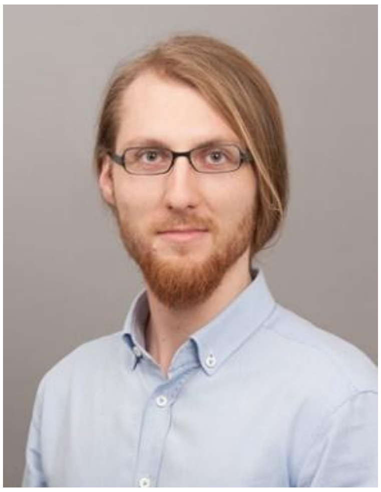

The importance of taxonomy and systematics (again) and some future opportunities for Trichoptera research

Taxonomy and systematics are fundamental to understanding biological diversity, ecological processes and ecosystem functioning. At the same time this discipline is at the basis of defining conservation targets, biomonitoring and assessment of biodiversity status through, e.g., Red Lists. Indeed, taxonomy and systematics are a crucial resource of societal relevance in the global biodiversity decline by providing the tools (authoritative species lists, identification keys, ecological metrics and reference libraries) to define biodiversity targets and monitor whether these are met. Unclear taxonomy and systematics preclude this as decision-making requires stable and reliable biodiversity data. However, there is yet no consensus regarding the scientific strategies to be used to resolve unclear taxonomy and systematics in taxonomic revisions. At the same time, taxonomic revisions are, to many aquatic entomologists, among the most exciting things, next to discovering new species and finding out what they actually do.

In this talk, I will use the Drusinae (Insecta, Trichoptera, Limnephilidae) – a group of caddisflies in which a taxonomic revision is clearly justified – as model to outline an integrative taxonomic workflow and argue how an authoritative species list could be obtained. Moreover, I will draw on other examples of freshwater biodiversity (Plecoptera, Mollusca: Unionidae) to demonstrate how important good taxonomy and systematics, and thus, taxonomic revisions, are.

I argue that taxonomic revisions can be best achieved by combining large-scale sampling efforts to provide fresh material for the majority of taxa, and completing the taxon sampling will be completed through loans from scientific collections. Experiments and phenological series should be used to assess intraspecific variation of taxonomic characters and to identify a set of relevant diagnostic taxonomic characters for integrative taxonomy. In a fully integrative taxonomic design, genome sequencing can be used to procure reference genomes for the target taxon and combined with shallow resequencing data to produce sets of comparable molecular data. Finally, I will outline a workflow using morphometrics and classic comparative taxonomy in combination with genomic data that could be used to test and delineate species hypotheses and propose tentative species lists. These should then be authorized by a large team of taxonomic specialists.

As side notes, I will outline other exciting opportunities for Trichoptera research and discuss how we could – maybe – approach them.

ABSTRACTS

18th International Symposium on Trichoptera
Quito, Educador, 1-5 July 2024

(arranged alphabetically by first author; presenting author in **bold**)

Notes on DNA barcoding of *Rhyacophila masudi*, Ali et. Al. 2024 (Trichoptera: Rhyacophilidae)

Tabraq Ali[1], Zahid **Hussain**[1], Aquib Majeed[1], Rifat Hussain Raina[2], and Sajad H. Parey[1]

1–*Insect Systematics Research Lab, Department of Zoology, Baba Ghulam Shah Badshah University, Rajouri, India* **2**–*Desert Regional Centre, Zoological Survey of India, Jodhpur, Rajasthan, India*

Corresponding author: Tabraq Ali (tabarakali89@gmail.com)

Rhyacophila masudi, was earlier described by Ali et. al. 2024 from the Northwest Himalaya of India based on morphological analysis. Now we are adding notes on DNA barcoding of this species. In this study, we conducted a comprehensive morphological and DNA barcoding analysis using the mitochondrial cytochrome c oxidase subunit 1 (COI) gene to investigate the taxonomic relationships and biodiversity of *Rhyacophila* species collected from various freshwater habitats across the North-Western Himalayas of India from 2019-2023. Besides the traditional taxonomy, we have recently switched to the molecular taxonomy of Trichoptera of India.

Aquatic macroinvertebrate diversity and water quality along an anthropic gradient in the upper Napo River Basin

Cyntia Daniela **Alvear Sayavedra**[1], Mariana Vellosa Capparelli[2], and Rodrigo Espinosa[3,4]

1–*Facultad de Ciencias de la Tierra y Agua, Universidad Regional Amazónica Ikiam, Tena Ecuador & Grupo de Biogeografía y Ecología Espacial – BioGeoE2, Universidad Regional Amazónica Ikiam, Tena, Ecuador* **2**–*Instituto de Ciencias Del Mar y Limnología-Estación El Carmen, Universidad Nacional Autónoma de México, Ciudad Del Carmen, México* **3**–*Facultad de Ciencias de La Vida, Universidad Regional Amazónica Ikiam, Tena, Ecuador* **4**–*Grupo de Biogeografía y Ecología Espacial – BioGeoE2, Universidad Regional Amazónica Ikiam, Tena, Ecuador*

Corresponding author: Cyntia Daniela Alvear Sayavedra (alvear714@gmail.com)

River ecosystems like the Napo River Basin (NRB), provide vital services but are threatened by anthropogenic activities such as resource extraction, deforestation, and unsustainable agriculture, thus highlighting the importance of basin-wide water quality monitoring. Aquatic macroinvertebrates and biotic indices are effective bioindicators for assessing environmental impact in the Neotropical Region. This study analyzed the diversity and composition of bioindicators in impacted areas of NRB. Thirty-six samples were collected from sites categorized as: Crop or Aquaculture (CA), Gold Mining (GM), Wastewater Discharge (WD), and Sites with Few Threats (FT). Macroinvertebrates and biotic indices such as AAMBI, BMWP-Col, and CCE were used to assess water quality. A total of 1218 macroinvertebrates shared among 20 orders, including Trichoptera, were identified. FT sites had the highest species richness and abundance, with significant differences compared to CA, GM, and WD sites. The AAMBI, BMWP-Col, and CCE indices indicated that FT sites had the best biological water quality, while GM and WD sites had poorer quality. The study reveals that human activities negatively impact water quality and biodiversity, emphasizing the need for stakeholders to act, especially in gold mining areas, and to develop sustainable farming practices to reduce pesticide runoff.

Trichopteran community changes across different land uses and diets of *Mortoniella* sp. (Trichoptera: Glossosomatidae) & *Smicridea* sp. (Trichoptera: Hydropsychidae) in Andean rivers

Andrés **Castañeda**, Christian Villamarín, and Blanca Ríos-Touma

Grupo de Investigación Biodiversidad, Medio Ambiente y Salud (BIOMAS), Facultad de Ingenierías y Ciencias Aplicadas. Ingeniería Ambiental, Universidad de Las Américas, Quito, Ecuador

Corresponding author: Esteban Andrés Castañeda Chávez (esteban.castaneda@udla.edu.ec)

Andean rivers exhibit unique environmental conditions, where river forest banks, land use, and type of basin collectively influence various biological processes. These characteristics may significantly impact the community composition and feeding habits of macroinvertebrates. This study investigates the trichopteran community composition associated with rocks, and the diets of two genera of Trichoptera (*Mortoniella* and *Smicridea*) across different land uses and basins. Eight rocks were selected in each sample site delineated by an 8x8 cm area on the upper surface of the rocks and were scraped of periphyton to analyze the feeding habits of the insects. Physical chemical parameters were measured. Our results show differences between physical-chemical characteristics between each land use type, but not in the community composition. Nonetheless, there are significant differences in the community composition at the basin scale. The gut content varies according to the dominant land use in the drainage area. These findings underscore the role of river habitat in shaping community dynamics and food availability. The results suggest that the river basin has a greater impact than land use.

Trichoptera in Singapore: A first look

Yee Qi **Chan**[1], Yuchen Ang[2], Bryna J.Y. Liang[1], Darren C.J. Yeo[1,2], John C. Morse[3]

1–*Department of Biological Sciences, Faculty of Science, National University of Singapore* **2**–*Lee Kong Chian Natural History Museum, Faculty of Science, National University of Singapore* **3**–*Department of Plant & Environmental Sciences, Clemson University, Clemson, South Carolina, USA*

Corresponding author: Yee Qi Chan (e0550327@u.nus.edu)

In land-scarce Singapore where fresh water is a critical resource, Trichoptera communities can serve as a biomonitoring tool, yet are poorly known. This study seeks to address this gap by establishing a preliminary understanding of trichopteran diversity and distribution in Singapore's freshwater streams. During October 2023–January 2024, 11 sites across nature reserves (five forest streams), buffer parks (four buffer streams), and urban areas (two urban streams) were surveyed for trichopteran larvae. Only eight sites (four in forest streams, four in buffer streams) yielded Trichoptera, totalling 107 larval specimens comprising five families (Calamoceratidae, Ecnomidae, Hydropsychidae, Leptoceridae, Odontoceridae). Numerically Leptoceridae dominated, with 54 individuals across six sites; Ecnomidae were the rarest, with only four individuals from three sites. Hydropsychidae were the most widely distributed, with 29 individuals from seven of the eight sites. Trichoptera were absent from the urban streams, which had greater depth and total dissolved solids, while similar Trichoptera assemblages and environmental parameters were recorded in both forest and buffer streams. Although differences were not significant, buffer streams had the highest abundance and taxonomic richness. These findings thus present a first look into Trichoptera in Singapore's streams, providing a baseline for further research and informing long-term freshwater conservation efforts.

Temporal and spatial comparative analysis of taxonomic richness and functional diversity of Trichoptera in urban and rural waters of middle and lower Cauca Basin, Colombia

Alejandra **Correa-Bedoya** and Fernando J. Muñoz-Quesada

Universidad de Antioquia, Medellín, Colombia

Corresponding author: Alejandra Correa-Bedoya (bioaleja0508@gmail.com)

Our study fills a crucial research gap by examining the effects of land use changes and habitat degradation on the spatial and temporal richness patterns and functional diversity of Trichoptera in the middle and lower Cauca basin, Colombia. Drawing on four years of comprehensive data (2019-2022) collected through an ongoing dam monitoring program and in-situ habitat characterization, we present a thorough analysis. By utilizing an adapted version of the Corine Land Cover methodology for Colombia and considering factors such as water usage and anthropogenic disturbance at the reach scale, we classified sites as either rural or urban. Our investigation included an assessment of various assemblage structure variables, including the number of genera, community composition, diversity estimators, percentage of collectors-filterers, percentage of predators, and percentage of shredders. Our findings revealed higher caddisfly richness values in rural water systems compared to urban ones, with peak taxonomic richness occurring between 2020 and 2021. These results highlight a distinct ecological diversity between rural and urban environments, emphasizing the complex relationship between land use changes, habitat degradation, and Trichoptera structure and composition in the Cauca basin.

Filling the Linnean and Wallacean gaps in Eastern Amazon: two new species of *Polyplectropus* Ulmer, 1905 (Trichoptera: Polycentropodidae) and new distributional records of other polycentropodids

Anne M. Costa[1], Laisse Moura[1], Leandro Juen[2], and Fábio B. **Quinteiro**[1]

1–*Laboratório de Estudos Comparativos em Insetos, Universidade Federal do Pará (UFPA), Instituto de Estudos Costeiros (IECOS), Bragança, Pará, Brazil* **2–***Laboratório de Ecologia e Conservação, Universidade Federal do Pará (UFPA), Instituto de Ciências Biológicas (ICB), Belém, Pará, Brazil*

Corresponding author: Fábio B. Quinteiro (fabiobquinteiro@gmail.com)

The Amazon biome stands as the paramount biodiversity hotspot globally. Despite its renowned richness, numerous species within this biome remain undescribed. Currently, the Brazilian Amazon has around 300 caddisfly species records. Within Polycentropodidae, only four genera have been recorded in the Brazilian Amazon: *Cernotina*, *Cyrnellus*, *Nyctiophylax*, and *Polyplectropus*, which presents 45 valid species. In an effort to enhance our understanding, we present the description of two new species of *Polyplectropus* and expand the distribution knowledge of five species of polycentropodids in the Brazilian Amazon. *Polyplectropus* new species 1 can be distinguished among the others in the *Polyplectropus fuscatus* Group by its intermediate appendage, in lateral view, with two membranous processes, digitate and directed dorsally. *Polyplectropus* new species 2 can be diagnosed from the other species in the *Polyplectropus bredini* Group by its dorsal and ventral branches of the inferior appendage with the same length, in lateral view, and the dorsal branch oblong, with distinct constriction at midlength. *Cernotina bibrachiata*, *C. flexuosa*, *C. uara*, *Cyrnellus rianus*, and *Polyplectropus alienus* are recorded for the first time in the state of Pará, eastern Amazon. These results underscore the existing gaps in our understanding of caddisfly taxa within critical biomes, like the Amazon.

Microhabitat use and seasonality of caddisflies (Trichoptera: Insecta) in two streams in eastern Cuba

Pedro López Del Castillo[1], Perla Alonso Eguía-Lis[2], Liliana María Gómez Luna[3], and Germán M. López Iborra[4] [presented by Monika Springer]

1–*Centro Oriental de Ecosistemas y Biodiversidad (BIOECO), Cuba* **2**–*Instituto Mexicano de Tecnología del Agua, Mexico* **3**–*Centro Nacional de Electromagnetismo Aplicado, Cuba* **4**–*Alicante University, Spain*

Corresponding author: Pedro López Del Castillo (pldelcastillo0@gmail.com)

During both rainy and dry seasons, we sampled four microhabitats in pools (cobbles, sand, leaf litter, and bank vegetation) and one microhabitat (cobbles) in riffles to identify groups of caddisfly species that share microhabitats in eastern Cuban streams. To explore the abundance of the caddisfly species by micro-habitats, a non-lineal clustering k-means method was used. This analysis allows to cluster the samples in seven groups according to the abundance by micro-habitats, seasonality, altitude and stream order. There were found 4338 individuals belonging to 11 families, 18 genera and 28 taxa (species and morphospecies). The results showed that the caddisfly abundance is strongly influenced by microhabitats, seasonality, and river characteristics. Riffles were the more abundant microhabitat independent of seasonality. This study provides important ecological information related to abundance patterns measured by microhabitats in mountain rivers with different anthropic pressure in eastern Cuba.

Diversification and historical biogeography of long-horned caddisflies (Trichoptera: Leptoceridae)

Everton S. Dias[1], Adolfo R. Calor[2], Albane **Vilarino**[3], Pitagoras C. Bispo[4]

1–*Departamento de Ciências da Natureza, Faculdade SESI SP de Educação, São Paulo, Brazil* **2**–*Laboratório de Entomologia Aquática, Instituto de Biologia, Universidade Federal da Bahia (UFBA), Salvador, Bahia, Brazil* **3**–*Departamento de Zoologia, Instituto de Biociências, Universidade de São Paulo (USP), São Paulo, São Paulo, Brazil* **4**–*Laboratório de Biologia Aquática, Departamento de Ciências Biológicas, Faculdade de Ciências e Letras, Universidade Estadual Paulista (UNESP), Assis, Brazil*

Corresponding author: Albane Vilarino (albanevilarino@alumni.usp.br)

Here, we examine temporal and spatial diversification patterns in the Leptoceridae, its ancestral distribution range, and the role of biogeographical events in the family diversification. A time-calibrated phylogeny of Leptoceridae was inferred using five molecular markers (COI, CAD, IDH, EF-1α and POL II) and including 38 of the 47 genera of the family (adding five genera to previous Malm & Johanson (2011) data sample: *Amazonatolica*, *Amphoropsyche*, *Leptecho*, *Neoathripsodes* and *Russobex*). Phylogenetic analysis was performed through Bayesian Inference in MrBayes 3.2.7, and divergence times were inferred in BEAST 2. Biogeographical analyses were performed in BioGeoBEARS. The time-calibrated phylogeny suggests the origin of Leptoceridae during the Lower Cretaceous (~135 MYA) in the supercontinent Gondwana and the highest diversification between Upper Cretaceous (~80 MYA) to Eocene (~35 MYA). The first cladogenesis (125 MYA), resulting in two sister groups (Leptorussinae + Triplectidinae, estimated to be ~115 MYA with ancestral range in East Gondwana) + (Grumichelinae + Leptocerinae, estimated to ~120 MYA with ancestral range in West Gondwana). These results are congruent with the Gondwana break up into two blocks during the Lower Cretaceous (~135 MYA).

eDNA in the Neotropics: Testing its efficacy as a biomonitoring tool

Isabella M. **Errigo**[1], Andrés Morabowen[2], Paul B. Frandsen[1], and Blanca Rios-Touma[2]

1– *Department of Plant and Wildlife Sciences, Brigham Young University, Provo Utah, USA* **2–***Grupo de Investigación Biodiversidad, Medio Ambiente y Salud (BIOMAS), Facultad de Ingenierías y Ciencias Aplicadas. Ingeniería Ambiental, Universidad de Las Américas, Quito, Ecuador*

Corresponding author: Isabella M. Errigo ime25@cornell.edu

Environmental DNA (eDNA) is an exciting molecular tool that pulls shed DNA from environmental samples to identify the organisms present. An increasingly popular method, eDNA has been shown to be cost effective and time efficient when compared to traditional biomonitoring methods in a variety of fields. Aquatic ecologists have begun to use eDNA as a tool to assess the biodiversity of aquatic macroinvertebrates in a system and quantify the water quality based on the presence/absence of contaminant-sensitive taxa. Although our understanding of eDNA is well developed throughout much of the Global North, in the Neotropics, we know very little about the efficacy and persistence of eDNA. To begin to fill this gap, we selected 18 rivers in the Ecuadorian Chocó and sampled both eDNA and kick samples monthly, for four months. An initial comparison showed that biodiversity metrics and the biological index scores of the two sampling methods did not align. However, further analysis using raw sequences revealed clear correlations that are hidden by information lost after taxonomic assignment. We conclude that, while there is promise for eDNA in the Neotropics, reference databases must be more complete before it can be used as a reliable monitoring tool.

What do genes have to do with caddisfly silk?

Paul B. Frandsen

Department of Plant and Wildlife Sciences, Brigham Young University, Provo, Utah, USA

Corresponding author: Paul B. Frandsen (paul_frandsen@byu.edu)

The underwater structures built by caddisflies have captured the imaginations of biologists for centuries. The key material shared by all caddisfly structures is an underwater, adhesive silk. This amazing viscoelastic biomaterial adheres to a variety of substrates making it an ideal mortar responsible for the myriad extended phenotypes of larval caddisflies. The ability to produce silk is shared with Lepidoptera; however, caddisfly silk is fundamentally different from most lepidopterans in that it is specially adapted to underwater use. Here, I will explore the evolution of the genes that encode for the primary caddisfly silk proteins and elaborate on how molecular adaptations in these genes interact with the aquatic environment to generate the variety of aquatic silk phenotypes we observe across caddisflies.

Milne and Milne revisited: The evolution of caddisflies

Paul B. **Frandsen**[1] and Ralph W. Holzenthal[2]

1–*Department of Plant and Wildlife Sciences, Brigham Young University, Provo Utah, USA* **2–***Department of Entomology, University of Minnesota, St. Paul, Minnesota, USA*

Corresponding author: Paul B. Frandsen (paul_frandsen@byu.edu)

In 1939, a classic paper on caddisfly larval evolution was published by Margery and Lorus Milne. In the paper, they drew some of the first "phylogenetic trees" for caddisflies, albeit without any formal statistical analyses. In the paper, they presented a remarkable illustration outlining their ideas on the evolution of caddisflies, based on larval behavior and habitats. Since their paper was written, the field of phylogenetics has been revolutionized with large, genome-wide molecular datasets becoming the norm and a large suite of statistical methods for inferring ancestral states. Here, we revisit the hypotheses of Milne and Milne 1939 and test them with a robust phylogeny and modern methods for ancestral state reconstruction.

Assessing trichopteran diversity in the Oglán River watershed: Insights from Amazonian Kichwa communities

Silvana **Gallegos-Sánchez**[1,2] and Iván Jácome-Negrete[1]

1–*Facultad de Ciencias Biológicas, Universidad Central del Ecuador, Campus El Dorado-Itchimbía, Quito, Ecuador* **2**–*ECOFORENSIC CIC*

Corresponding author: Silvana Andrea Gallegos Sánchez (yantana.sags@gmail.com)

The Oglán River flows through a small watershed where several Amazonian Kichwa communities reside in their ancestral territory. Much of this territory comprises pristine tropical rainforests with unpolluted streams and rivers. However, anthropogenic pressures such as oil extraction and logging pose significant threats to this area, where knowledge about Trichopteran diversity is limited. To address this gap, we initiated a citizen science project involving the Pablo López del Oglán Alto and the Elena Andy del Uklan Yaku Communities. Together, we actively sampled five streams and the Oglán River to assess the diversity of aquatic macroinvertebrates. We identified and categorized Trichoptera from other orders and analyzed the community composition. In total, we collected 1333 aquatic invertebrates, of which 15% (197) were caddisfly larvae. We identified 10 Trichopteran families and 17 genera. The most abundant family was Hydropsychidae, followed by Leptoceridae. Among the sampled rivers, the Yurak Oglan stream exhibited the highest diversity (H'=2.25). In Kichwa indigenous knowledge, all trichopteran taxa were referred to as "Rumi Shundu." Hydropsychidae larvae were primarily utilized as fishing bait.

Functional traits of the ancestral caddisfly larva

Xinyu Ge[1] and John C. **Morse**[2]

1–*Tianjin Key Laboratory of Conservation and Utilization of Animal Diversity, College of Life Sciences, Tianjin Normal University, Tianjin, P.R. China* **2**–*Department of Plant and Environmental Sciences, Clemson University, Clemson, South Carolina, USA*

Corresponding author: John C. Morse (jmorse@clemson.edu)

Recent phylogenomic studies have concluded that the ancestor of suborder Integripalpia and order Trichoptera probably had a larva that was "free living," without a portable case or fixed retreat (Frandsen et al. in press, Ge et al., in review). We reflect further on the phylogenies inferred from those investigations with hypotheses regarding other probable functional traits of larvae of the Trichoptera ancestor and its immediate descendants and of the extant amphiesmenopteran sister lineage Lepidoptera. Like the ancestral moth larva, the ancestral caddisfly larva was not only "free living," but also was often submerged and with movement enhanced by a hook on each anal proleg. The ancestral integripalpian larva constructed a precocious dome-like pupal shelter (= portable case) in instar V. The hydroptiloid ancestor underwent hypermetamorphosis and constructed a purse-shaped case. Larvae of the glossosomatoid-rhyacophiloid ancestor lived in lotic habitats and spun semipermeable cocoons. Meanwhile, the ancestral annulipalpian larva constructed a fixed retreat with an upstream filternet in lotic habitats on exposed surfaces of stable substrates in fast water.

Using submerged light traps to learn about caddisflies that dive to oviposit around the world

William Gerth[1], Christina Murphy[2], Ivan Arismendi[1], and Tatiana **Latorre-Beltran**[1]

1–*Department of Fisheries, Wildlife, and Conservation Sciences, Oregon State University, Corvallis, Oregon, USA* 2–*U.S. Geological Survey, Maine Cooperative Fish and Wildlife Research Unit, University of Maine, Orono, Maine, USA*

Oviposition is a critical step in the life cycles of aquatic insects. Adult caddisflies exhibit a variety of oviposition methods. In some species, females dive and swim to oviposit on submerged substrates. In the process of sampling for another purpose, we found that submerged light traps can be an effective way of catching adult female caddisflies as they dive and swim to lay eggs. We have also summarized published information on North American species that dive and swim or have leg modifications that would presumably facilitate swimming and documented the additional species we captured in submerged light traps. In North America, 24 caddisfly species are known to have females that dive and swim to oviposit and 41 species are recorded as having leg modifications that suggest they could swim. Most are in the family Hydropsychidae plus a few in other annulipalpian families, but there are also diving species in the families Hydroptilidae and Phryganeidae, and leg modifications that would suggest swimming ability in the Glossosomatidae. We would like to collaborate with colleagues around the world to deploy submerged light traps to document additional caddisfly species that dive and swim for egg laying.

Elevational diversity of *Rhyacophila* (Trichoptera: Rhyacophilidae) in the Hengduan Shan

Sonja Gerwin, Xiling Deng, and Steffen U. **Pauls**

Senckenberg Research Institute and Natural History Museum Frankfurt, Germany

Steffen U. Pauls (steffen.pauls@senckenberg.de)

Aquatic insects are particularly sensitive to disturbances in aquatic habitats because their life cycles are directly linked to physico-chemical conditions in these environments. Here we combine molecular tools and ecological analysis to determine general distributional patterns and associated environmental variables for an unknown fauna of *Rhyacophila* caddisflies in the Hengduan Mountains in China. In total 415 larval and 109 adult specimens from four river catchments between 1022 m - 4381 m a.s.l. were used for the analyses. Tree-based and distance-based methods were used to delimit putative molecular operational taxonomic units (MOTUs) based on both mitochondrial COI (mtCOI) and nuclear wingless (nuWG) data. Overall, 33 adult males and 14 females were successfully associated to larvae. Many MOTUs were geographically restricted and rare, often occurring only in one or two sampling sites. PCA and multivariate GLM analyses confirmed the importance of altitude and chemical stream variables in explaining the abundance of many larval *Rhyacophila* MOTUs. In combination, the strong explanatory power of altitude and high level of geographical restriction of MOTUs indicates that *Rhyacophila* in the Hengduan Shan are vulnerable to ongoing climate change.

Relationship of Trichoptera species in Iceland with other North-Atlantic islands and the mainland of Europe

Gísli Már **Gíslason** and Snaebjörn Pálsson

Institute of Life and Environmental Scineces, University of Icelan, Reykjavík, Iceland

Corresponding author: Gísli Már Gíslason (gmg@hi.is)

Geographic variation in the COI mtDNA barcode marker in ten of the Trichoptera species from Iceland indicates distinct histories where different species show indication of varying time since colonization of the island and separate evolution restricted to Iceland. One of the three Holarctic species, the parthenogenic

Apatania zonella, appears to have originate near the Bearing Strait and dispersed west to Scandinavia and diverged into a separate lineage within Iceland, where another route was east N-America to Greenland and the populations met in Iceland. *Limnephilus fenestratus* and *L. picturatus* do not show a clear split between the Nearctic and Palearctic. Four of the palearctic species *L. affinis*, *L. griseus*, *L. sparsus*, *L.elegans* present unique lineages within Iceland, suggesting an early colonizeration after the Ice-age. Variation within the three other species reflect a recent origin. *Potamophylax cingulatus* originates in Central Europe and its variation reflects a migration route west to France and then north along the coast to Britain and finally to Iceland in the 20th century. *Limnephilus decipiens* and *Microptsectra sequax*, a very recent colonizer (21st century) fall genetically in with other European species. Five of the ten species suggest unique mtDNA lineages within Iceland suggesting an early colonization.

The Trichoptera of Ecuador

Ralph W. **Holzenthal**[1] and Blanca Ríos-Touma[2]

1–*Department of Entomology, University of Minnesota, St. Paul, Minnesota, USA* **2**–*Grupo de Investigación Biodiversidad, Medio Ambiente y Salud (BIOMAS), Facultad de Ingenierías y Ciencias Aplicadas. Ingeniería Ambiental, Universidad de Las Américas, Quito, Ecuador*

Corresponding author: Ralph W. Holzenthal (holze001@umn.edu)

Ecuador, crossed by the equator and bisected by the Andes, harbors a remarkable diversity of plant and animal species. For example, 1663 bird species have been recorded from the country compared to 1023 for all North America. This diversity is reflective of the northern Andean region of South America as a whole, probably the most biodiverse region on the globe. What is the caddisfly diversity of Ecuador? To address this question, we have been continuously exploring the fauna since 2011. To date, we have recorded 493 species from the country based on 49,914 curated and databased specimens from 265 collection events. Of these, 25 new species and one new genus have been described and 172 new or potentially new species await study and description. The current total of species is 183 more than recorded in our first assessment of the fauna published in 2017, representing more intensive field work since 2020. Chao 2 species richness estimator based on species incidence data predicts that the actual fauna contains 689 species. In addition to more survey work, future efforts will include COI barcode sequencing for each species and continued research into speciation processes and ecological adaptations based on genetic data

Analyzing adult Trichoptera to assess upstream disturbance: one (caddis) metric to rule them all?

David C. Houghton

Department of Biology, Hillsdale College, Hillsdale, Michigan, USA

Corresponding author: David C. Houghton (dhoughton@hillsdale.edu)

Adults of Trichoptera are valuable for assessing riverine biotic integrity; however, it is not clear which specific metric(s) are most effective for doing so. In this study, >600,000 adult caddisflies were sampled from 808 stream sampling sites throughout the northcentral United States. Specimen data were compiled into 24 water quality metrics encompassing taxonomic richness, diversity indices, functional traits, pollution tolerance, and organic biomass. Each metric was tested for its ability to predict the known level of undisturbed habitat upstream of each sampling site using simple linear regression modeling. Twenty models were significant and seven had R2 >0.25. Undisturbed upstream habitat increased with percentage of shredder biomass (0.27), ratio of shredders to filtering collectors (0.28), and richness at the species (0.41), genus (0.53), and family (0.61) levels, and decreased with percentage of filtering collector biomass (0.34) and the Hilsenhoff Biotic Index (0.37). A multiple linear regression analysis of all 24 metrics produced a model that explained only 8% more dataset variation than did family richness exclusively. These results indicate that taxonomic richness metrics constitute the most effective predictors of undisturbed upstream habitat, and that family richness may be the most valuable due to its ease of use and low stochastic variation.

Taxonomy and DNA barcodes of the family Philopotamidae (Trichoptera: Insecta) from India

Zahid **Hussain**[1], Aquib Majeed[1], Tabraq Ali[1], Sajad H. Parey[1], and Manpreet S. Pandher[2]

1–*Insect Systematics Research Lab, Department of Zoology, School of Biosciences and Biotechnology, Baba Ghulam Shah Badshah University, Rajouri, India* **2**–*High Altitude Regional Centre, Zoological Survey of India, Saproon, Solan, Himachal Pradesh, India*

Corresponding author: Zahid Hussain (zahidshah2@gmail.com)

The family Philopotamidae, commonly known as finger-net caddisflies, under the suborder Annulipalpia, is represented by 1508 species under 26 genera all over the Globe and 625 species are inhabitants in the Oriental region. In India, the family is represented by 7 genera and 155 species. These organisms are significant bioindicators of water quality and are crucial in the aquatic food web. Despite their ecological importance, the taxonomy and molecular profiling of Philopotamidae in India remain inadequately explored. This study aims to fill this gap by providing a comprehensive taxonomic revision and DNA barcode assessment of the Philopotamidae family in India. This study provides the first detailed taxonomic revision and DNA barcode analysis of the Philopotamidae family in India, contributing to the global efforts in cataloging biodiversity and understanding evolutionary relationships within Trichoptera. In the present study, we have identified seven species of Philopotamidae with notes on their DNA barcodes. The complete taxonomic overview and phylogenetic analysis of the species shall be presented during XVIIIth Intranational Symposia on Trichoptera at Ecuador.

First data on Trichoptera of the Putorana Plateau (Northern Siberia)

Vladimir **Ivanov**[1], Stanislav Melnitsky[1], and Andrey A. Przhiboro[2]

1–*Department of Entomology, Saint-Petersburg State University, St. Petersburg, Russia* **2**–*Laboratory of Freshwater and Experimental Hydrobiology, Zoological Institute of Russian Academy of Sciences, St. Petersburg, Russia*

Corresponding author: Vladimir D. Ivanov (v.d.ivanov@spbu.ru)

The Putorana Plateau is a mountain massif up to 1.7 km a.s.l. situated at 67–71°N, south of the Taymyr Peninsula in northwestern part of the Central Siberian Highlands. It is formed by basalts from the Siberian Traps. The plateau is cut by deep valleys with rivers and lakes. The Putorana region has a high diversity of biotopes and comprises three landscape zones: taiga, predominantly larch forest in valleys, mostly below 600 m, mountain tundra generally between 600 and 1000 m, and arctic desert usually above 900 m. Over one thousand adult and immature caddisflies were collected by the latter author in 2019–2023 from several hundred localities in nine areas of the Putorana Plateau. More than 25 species were identified; some of them seem to be new for science. Most of the recorded species belong to the suborder Integripalpia with dominant families Apataniidae and Limnephilidae. Other families are Polycentropodidae, Glossosomatidae, Phryganeidae, and Brachycentridae. The fauna is peculiar in the absence of filter-feeding lotic species and the complete absence of the Brevitentoria families. Polycentropodidae, Hydroptilidae and Glossosomatidae are represented by a single species each. Males of the predominantly parthenogenetic species *Apatania forsslundi* were found for the first time.

Morphology outperforms DNA barcoding in identifying Trichoptera in Ecuadorian streams

Gabriela **Jijón**[1,2], Isabella M. Errigo[1,2,3], Jessica Wicks[1], Natalie Nyborg[1], Lillian Buck[1], Daniel Davis[1], Sam Standring[1], John Chaston[1], Blanca Rios-Touma[2], Paul B. Frandsen[1]

1–*Department of Plant and Wildlife Sciences, Brigham Young University, Provo, Utah, USA* **2–***Grupo de Investigación en Biodiversidad, Medio Ambiente y Salud (BIOMAS), Facultad de Ingenierías y Ciencias Aplicadas, Universidad de Las Américas, Quito, Ecuador* **3–***Department of Natural Resources, Cornell University, Ithaca, New York, USA*

Corresponding author: Gabriela Jijón (m.gabriela.jijon@gmail.com)

Using macroinvertebrate bioindicators is increasingly critical given the high levels of disturbance experienced in aquatic environments, particularly in the Neotropics. Such bioassessment studies have primarily characterized macroinvertebrate communities using traditional morpho-taxonomic identification approaches. With the rapid development of sequencing technologies, DNA-based approaches might allow more efficient identification of specimens in stream surveys. However, characterizing some groups with molecular-based approaches may be more challenging than others. For example, the high diversity of Trichoptera and low sequence representation of Neotropical species in the reference barcode databases can prevent accurate classification of their DNA barcodes. We performed a comparative study in Ecuadorian streams to assess the effectiveness of molecular-based approaches against the traditional characterization method of macroinvertebrate communities. Replicate samples were collected in ten sites across an elevation gradient in Ecuador and compared using morpho-taxonomic and metabarcoding approaches. We found low taxonomic overlap at family and genus ranks across sites for the macroinvertebrate communities, potentially due to a lack of reference species in the barcode databases and primer bias. Notably, lower Trichoptera richness was documented in the DNA approach compared to the morphological. Considering this, I will assess the state of Ecuadorian caddisfly barcodes in databases, identify gaps, and recommend improvements.

Contribution to the fauna, distribution, and DNA barcoding data of caddisflies (Insecta: Trichoptera) in Croatia

Mladen Kučinić

Department of Biology, Faculty of Science, University of Zagreb, Zagreb, Croatia

Corresponding author: Mladen Kučinić (mladen.kucinic@biol.pmf.hr)

The Republic of Croatia is geographically located in the central and southern part of Europe, where it belongs to the Central European and Mediterranean countries. It has a surface area of 56,000 km^2. Based on the climatological, geological, and geographical characteristics of the Croatian territory, three main regions can be distinguished: the Pannonian-Peripannonian area, the mountainous central area, and the Mediterranean area. Caddisfly studies began in Croatia at the beginning of the 19th century. So far, 215 species of caddisflies have been recorded in the fauna of Croatia, with more than 100 species recorded in all three biogeographical regions. During investigations in the last ten years, several new species of caddisflies have been found or described in the fauna of Croatia, e.g.: *Rhyacophila delici* Kučinić & Valladolid, *Agapetus kampos* Oláh, *Tinodes antonioi* Botosaneanu & Taticchi-Viganò, *Hydroptila simulans* Mosely, *Oxyethira falcata* Morton, *Ecclisopteryx asterix* Malicky, *E. ivkae* Previšić, Graf & Vitecek, and *Stenophylax mitis* McLachlan. In addition to several new species, about 180 species of Trichoptera from the Croatian fauna have been DNA barcoded to date, with 600 DNA barcoded specimens submitted to the BOLD database. Faunistic research and DNA barcoding of Croatian caddisflies will continue in the coming years.

Higher predicted climate-change vulnerability for spring-dwelling freshwater biota

Mathias Kümmerlen[1,2] and Steffen U. **Pauls**[3]

1–*Senckenberg Biodiversity and Climate Research Centre, Frankfurt am Main, Germany* **2**–*Bundesamt für Naturschutz, Bonn, Germany* **3**–*Senckenberg Research Institute and Natural History Museum Frankfurt, Frankfurt am Main, Germany*

Corresponding author: Steffen Pauls (steffen.pauls@senckenberg.de)

Environmental change threatens freshwater biodiversity through altered temperature and precipitation patterns. However, knowledge and data are frequently insufficient to determine impacts at the species level, leading to misinterpreted species vulnerability. Conversely, phylogenetic relationships, current distributions and ecological traits of the caddisfly subfamily Drusinae are well known. Thus, we assessed individual and trait-specific climate change (CC) vulnerability for 47 Drusinae species, by setting up species distribution models (SDMs). Species were grouped by larval feeding guild, stream zonation preference and level of endemism. Models were calibrated with predictors describing climate, topography and geology at a spatial resolution of 1 km^2 and were projected for five general circulation models under four future climate scenarios. To limit dispersal, distribution projections were restricted to a maximum of 500 km until the year 2080. Relative predicted range change fluctuated between -100% and 197%, with extinction predicted for five species. Altitudinal shifts varied between -2% and +15%, with distribution centroids shifting between 28 km and 119 km. Our results identify stream zonation, a non-phylogenetic trait, as the best indicator of CC vulnerability. Further, two important conclusions are highlighted: monitoring is best done at the species level, while the biodiversity of springs and low order streams requires considerably more research.

Trichoptera dispersion: insights from lateral and longitudinal sampling

Tatiana **Latorre Beltrán**[1], William Gerth[1], Ivan Arismendi[1], and Blanca Ríos-Touma[2]

1–*Department of Fisheries, Wildlife, and Conservation Sciences, Oregon State University, Corvalis, Oregon, USA* **2**–*Grupo de Investigación Biodiversidad, Medio Ambiente y Salud (BIOMAS), Facultad de Ingenierías y Ciencias Aplicadas. Ingeniería Ambiental, Universidad de Las Américas, Quito, Ecuador*

Corresponding author: Ivonne Tatiana Latorre Beltrán (ivonne-tatiana.latorre-beltran@oregonstate.edu)

Adult aquatic insects play a vital role in supporting aquatic and terrestrial food webs, with their dispersion and emergence influencing higher trophic levels in adjacent ecosystems. Given the abundance and diversity of Trichoptera, study of their dispersal patterns contributes to understanding the link between aquatic and adjacent systems. Here, we sampled adult aquatic insects in the HJ Andrews experimental forest in western Oregon, USA, to investigate lateral and longitudinal dispersion patterns. For the 2023 summer, six double rows of sticky traps were distributed within the main channel of the stream and in the riparian zone. Each trap was directed upstream, downstream, left, and right. We repeated this experiment six times in three streams for lateral dispersion and one for longitudinal. Also, five Malaise traps were set in one site. We observed five caddisfly genera dispersing laterally away from streams, with *Micrasema* exhibiting the greatest dispersion distance. Within the EPT group, Trichoptera comprised 68.5% of the capture in instream traps, showcasing a preference for upstream-downstream movement over lateral movement. *Rhyacophila* and *Dolophiloides* were the most abundant, dispersing longitudinally. Our findings contribute insights into the dispersion dynamics of aquatic insects, highlighting the prevalence of Trichoptera and the differential movement patterns observed longitudinally.

Species diversity of caddisflies (Insecta: Trichoptera) from lowland forest springs, Surat Thani Province, southern Thailand

Pongsak **Laudee** and Pimpajee Kaewwong

Faculty of Innovative Agriculture and Fishery Establishment Project, Prince of Songkla University, Surat Thani Campus, Muang District, Surat Thani Province, Thailand

Corresponding author: Pongsak Laudee (pongsak.l@psu.ac.th)

Cool springs and their streams of lowland tropical rainforests are unique ecosystems fed by groundwater outflow. The sediment of the cool springs is mainly sand and leaf detritus. Four cool springs in lowland tropical rainforest of Surat Thani Province, southern Thailand were chosen. Larvae and adults of caddisflies were collected with aquatic nets for larvae, and screen and pan light traps for adults. The preliminary result showed that *Paduniella sampati*, *Ecnomus quordaio*, *Triaenodes themis*, and *Oecetis biramosa*, are the most abundant species in these cool springs.

Caddisfly species identification via wing morphometrics

Stella Li, Colette Christensen, and Patina K. **Mendez**

Department of Environmental Science, Policy, and Management, University of California, Berkeley, Berkeley, California, USA

Corresponding author: patina.mendez@berkeley.edu

Species identification is required for biological and conservation applications but remains challenging in entomology where specimens are small and require expert skills for identification. Geometric morphometrics may be an underexplored method for species identification but has been used in the literature alongside machine learning to identify botanical scans of leaves to species. Using bulk light trap samples from Curry Creek in Northern California, we scanned mounted wings of males and females from the caddisfly families Sericostomatidae and Hydropsychidae, identifying 38 and 45 landmark locations, respectively. Using Generalized Procrustes Analysis (GPA) and Principal Component Analysis (PCA) we determined differences in venation between species and sex. GPA identified the central crossveins in Sericostomatidae as having the most variation throughout all specimens for both fore and hind wings, and PCA highlighted sexual dimorphism, particularly in the central crossveins, where males had more reduced wing venation than females. For Hydropsychidae, tests likewise revealed variation largely in the central crossveins; PCA scores quantified at least 50% variation between wing landmarks, enough to distinguish species into three distinct clusters for the site.

DNA barcoding and taxonomic insights of subfamily Hydropsychinae (Hydropsychidae: Trichoptera: Insecta) from India

Aquib Majeed[1], Tabraq Ali[1], Zahid **Hussain**[1], Sajad H. Parey[1], and Manpreet S. Pandher[2]

1–I*nsect Systematics Research Lab, Department of Zoology, School of Biosciences and Biotechnology, Baba Ghulam Shah Badshah University, Rajouri, India* **2**–*High Altitude Regional Centre, Zoological Survey of India, Saproon, Solan, Himachal Pradesh, India*

Corresponding author: Sajad H. Parey (sajadzoo@gmail.com)

The Hydropsychinae subfamily, belonging to the Hydropsychidae family of Trichoptera, represents a significant component of freshwater macroinvertebrate fauna in India. Despite its ecological importance, the taxonomic identification of Hydropsychinae species in India has been largely based on traditional morphological characteristics, which can be challenging due to the high morphological variability within the subfamily. The application of DNA barcoding provides a promising molecular approach to complement traditional taxonomy and enhance species identification accuracy. Our analysis revealed distinct barcode clusters for different

Hydropsychinae species, indicating the efficacy of DNA barcoding in discriminating and identifying species within this subfamily in the Indian context. In this study, we conducted a comprehensive morphological and DNA barcoding analysis using the mitochondrial cytochrome c oxidase subunit 1 (COI) gene to investigate the taxonomic relationships and biodiversity of Hydropsychinae species collected from various freshwater habitats across India from 2019-2023. We have generated barcodes of 49 specimens of Hydropsychinae. Additionally, the taxonomic insights gained from the DNA barcoding data facilitated a better understanding of the phylogenetic relationships and evolutionary history of Hydropsychinae species in India. Complete taxonomic description, DNA barcode data and phylogenetic relationship of the family Hydropsychidae in India shall be presented.

Growth and development of undergraduate Trichoptera researchers

Patina K. Mendez

Department of Environmental Science, Policy, and Management, University of California, Berkeley, Berkeley, California, USA

Corresponding author: Patina Mendez (patina.mendez@berkeley.edu)

Team Caddis is a group of undergraduate researchers at the University of California, Berkeley who work collaboratively on small-scale, iterative research questions using caddisflies as their primary model organism. Team members include students with a wide range of experience and skills from novice researchers to senior thesis students. The research and learning space centers on interdependent cultural norm framings hypothesized to signal inclusion and belonging for students of historically underrepresented backgrounds and increase persistence in STEM. Over the past 10 years, ~70 undergraduate members of Team Caddis have contributed to museum and ecological studies including: developing methods for imaging fluid-based Trichoptera collections, sorting and identification of monthly light trap samples from an intermittent stream in California, species distribution models of California Caddisfly genera, exploring wing venation for species associations of adult caddisflies using geometric morphometrics, and developing species description and illustration pages for the 300+ species of California caddisflies. Members of Team Caddis participate for ~2.5 semesters, experience an increased sense of belonging in research spaces, learn to collaborate, and develop research skills.

"Sense of Presence"

(special photographic presentation)

Rogier Maaskant

Rotterdam, The Netherlands; contact@rogiermaaskant.com

"The tiny creatures that run the world," is how myrmecologist Edward O. Wilson described insects. They are nature's cleaners, food for many other species and are essential for the pollination of plants, including our crops. For years, their populations have been plummeting dramatically due to habitat loss and the use of pesticides. It is vital that we stay connected with them and that is why they deserve a stage to put their importance and beauty in the limelight. Rogier Maaskant's enchanting images unveil a diverse array of insects in flight during twilight and nighttime – in forests and above fields, ditches, and ponds around Rotterdam, Netherlands. By using strobe light, his single exposures provide a unique view of the flight of caddisflies, moths, lacewings, dragonflies, gnats, and various other species that illustrate the rich biodiversity. For *Sense of Presence*, Maaskant collaborated with scientists and insect experts. His work is in line with the current era of renewed interest in nature and a widespread awareness of the need to protect it.

Landuse and elevation-driven changes in caddisfly assemblages in Neotropical streams in Ecuador

Andrés **Morabowen**[1], Blanca Rios-Touma[1], Isabella M. Errigo[1,2,3], and Paul B. Frandsen[2]

1–Grupo de Investigación en Biodiversidad, Medio Ambiente y Salud (BIOMAS), Facultad de Ingenierías y Ciencias Aplicadas, Universidad de Las Américas, Quito, Ecuador **2–***Department of Plant and Wildlife Sciences, Brigham Young University, Provo, Utah, USA* **3–***Department of Natural Resources, Cornell University, Ithaca, New York, USA*

Corresponding author: Andrés Morabowen Mantilla (andresmorabowen@gmail.com)

Freshwater ecosystems in Ecuador face ubiquitous threats, and conservation strategies should focus on the connectedness of different altitudinal ecosystems. To assess the uniqueness of freshwater macroinvertebrate communities along an altitudinal gradient, we sampled caddisfly larvae at two different altitudes (1800 and 550 m a.s.l.). We examined the Trichoptera assemblages, abundance, and distribution across 18 streams undergoing various levels of land-use modification at two different altitudes. The two altitudinal gradients, Nanegalito (1800 m a.s.l.) and Mashpi (500 m a.s.l.) are in the Andean Chocó ecoregion, a biodiversity hotspot. We found significant differences associated with changing environmental conditions at the two altitudinal sampling sites driven by specific genera. Our findings highlight the uniqueness of the freshwater Trichoptera communities along the Andean mountain range. Also, assemblages at the two different elevations responded to different environmental stressors. In the upper basins ammonia was the main stressor influencing differences in the Trichoptera community, and in the lower rivers, flow and oxygen saturation were responsible for differences among sites. Thus, we urge the implementation of management practices that support consistent conservation efforts across the entire altitudinal gradient. The connectivity of these habitats is crucial for preserving genetic diversity, thereby increasing ecological resilience and enhancing ecosystem services.

Larval taxonomy of the net-spinning caddisfly *Cernotina truncona* Ross, 1947 (Trichoptera: Polycentropodidae)

Alexander **Orfinger**[1], Truc Bui[1], Andrew Rasmussen[2]

1–*Department of Life Science, Dalton State College, Dalton, Georgia, USA* **2–***Center for Water Resources, Florida A&M University, Tallahassee, Florida, USA*

Corresponding author: Alexander Benjamin Orfinger (aorfinger@daltonstate.edu)

The polycentropodid genus *Cernotina* consists of diminutive caddisflies, with the largest individuals reaching maximum lengths of 8-9 mm. Of the seven Nearctic species, only the larva of *C. spicata* Ross, 1938 has been described and illustrated to date. *Cernotina spicata* is one of just three species of the genus recorded from the southeastern United States, the others being *Cernotina calcea* Ross, 1938 and *Cernotina truncona* Ross, 1947. Using ecologically and geographically associated larval specimens, we describe and diagnose the larva of *C. truncona*, noting subtle but consistent differences from *C. spicata*.

Exploring the Trichoptera in the Taxonomic Catalog of the Brazilian Fauna: recent progress and perspectives

Fabio B. **Quinteiro**[1], Adolfo R. Calor[2], Gleison R. Desidério[3], Leandro L. Dumas[4], Ana Lucia Henriques-Oliveira[5], Rafael Pereira[6], Ana Maria Pes[7], Allan P.M. Santos[8], and Albane Vilarino[9]

1–*Laboratório de Estudos Comparativos em Insetos, Universidade Federal do Pará (UFPA), Instituto de Estudos Costeiros (IECOS), Bragança, Pará, Brazil* **2–***Laboratório de Entomologia Aquática, Instituto de Biologia, Universidade Federal da Bahia (UFBA), Salvador, Bahia, Brazil* **3–***Programa de Apoio à Fixação de Jovens Doutores no Brasil (PROFIX-JD), Laboratório de Citotaxonomia e Insetos Aquáticos (LACIA), Instituto*

Nacional de Pesquisas da Amazônia (INPA), Manaus, Amazonas, Brazil **4–***Laboratório de Insetos Aquáticos (LABIA), Departamento de Biologia Animal, Universidade Federal Rural do Rio de Janeiro (UFRRJ), Seropédica, Rio de Janeiro, Brazil* **5–***Laboratório de Entomologia, Instituto de Biologia, Universidade Federal do Rio de Janeiro (UFRJ), Cidade Universitária, Rio de Janeiro, Brazil* **6***–Laboratório de Entomologia Aquática, Instituto de Biologia, Universidade Federal da Bahia (UFBA), Salvador, Bahia, Brazil* **7–***Programa de Apoio à Pós-Doutores (PRODOC), Laboratório de Citotaxonomia e Insetos Aquáticos (LACIA), Instituto Nacional de Pesquisas da Amazônia (INPA), Manaus, Amazonas, Brazil* **8–***Laboratório de Sistemática de Insetos, Universidade Federal do Estado do Rio de Janeiro (UNIRIO), Instituto de Biociências, Rio de Janeiro, Rio de Janeiro, Brazil;,* **9–***Departamento de Zoologia, Instituto de Biociências, Universidade de São Paulo (USP), São Paulo, São Paulo, Brazil*

Corresponding author: Fábio B. Quinteiro (fabiobquinteiro@gmail.com)

The Taxonomic Catalog of the Brazilian Fauna (CTFB) has been active since December 2015, providing comprehensive information on Brazil's animal fauna. In the 2020 checklist, Trichoptera comprised 728 species, making up 4.9% of the global caddisfly diversity, with Odonata ranking higher at 867 species. Brazil boasts approximately 934 Trichoptera species records (5.4% of the global caddisfly diversity), with 635 being endemic, surpassing Odonata's count of 910 species. This makes Trichoptera the most diverse aquatic insect order in Brazil. The Atlantic Forest remains the most-species rich biome, with 521 species (377 of them endemic), followed by the Amazon, with 306 species (207 of them endemic). Hydroptilidae remains the most diverse family, with 205 species, of which 23% were described in the last four years. The rise in species count might be attributed to the increasing caddisfly taxonomy research groups (currently at least six major groups) across three Brazilian states, involving the second and third generations of Brazilian caddisfly taxonomists. With an average description rate of 28.8 species/year from 2015–2024, the estimated Brazilian caddisfly diversity of 1,710–1,828 species could be uncovered within 59.4–63.5 years. The next phase involves incorporating distribution data to create online distribution maps.

Microcaddisflies (Trichoptera: Hydroptilidae) of the Baja California peninsula, Mexico, and their biogeographic affinities.

Mauricio **Ramírez-Carmona**[1], Atilano Contreras-Ramos[2], and Robin E. Thomson[1]

1–*Department of Entomology, University of Minnesota, St. Paul, Minnesota, USA* **2–***Departamento de Zoología, Instituto de Biología, Universidad Nacional Autónoma de México, México City, Mexico*

Corresponding author: Mauricio Ramírez-Carmona (ramre008@umn.edu)

Desert habitats dominate the Baja California peninsula, yet they contain remnant aquatic systems (oases) representing isolated islands of biodiversity. Previous faunal surveys have reported the distribution of 11 caddisfly families in the region, but the family Hydroptilidae has not been documented (Dennig, 1964). A single microcaddisfly species has been reported from the southern Baja peninsula (Razo-González, 2023), yet the overall caddisfly fauna of this area remains understudied. This study presents the first microcaddisfly (Hydroptilidae) diversity assessment in the Baja California peninsula. Fieldwork conducted over two years (2021-2022) resulted in the identification of 5 genera: *Hydroptila*, *Leucotrichia*, *Neotrichia*, *Ochrotrichia*, and *Oxyethira*. In total, 15 hydroptilid species corresponding to these genera are reported, significantly expanding the known diversity of this group. Also, an area cladogram analysis was performed to evaluate the biogeographic affinities of the Baja California peninsula. These findings contribute to understanding the composition and distribution of Hydroptilidae within this isolated desert landscape, representing an understudied component of the regional aquatic insect fauna. The results provide a crucial baseline for future biodiversity assessments and biogeographic studies in the Baja California peninsula.

Towards a robust phylogeny of Stactobiinae (Trichoptera: Hydroptilidae): synthesizing molecular and morphological data

Mauricio **Ramírez-Carmona** and Robin E. Thomson

Department of Entomology, University of Minnesota, St. Paul, Minnesota, USA

The subfamily Stactobiinae (Hydroptilidae) was established by Botosaneanu (1956) and currently includes 12 genera and 481 species distributed across the Holarctic, Indomalayan, Neotropical, and Afrotropical regions. Despite efforts to taxonomically define the group, several authors highlight the substantial morphological variation within the subfamily, posing challenges in establishing a unified classification (Marshal, 1979; Wells, 1990; Thomson, 2023); questions persist regarding its monophyly, biogeography, and morphology. Furthermore, phylogenetic relationships among Stactobiinae taxa have been addressed in only a few studies, and no statistical analysis has yet been used in these works. The purpose of this study is to establish a comprehensive phylogeny using molecular data, particularly targeted enrichment data, and morphological data, as well as to establish the morphological criteria defining the taxa of the subfamily Stactobiinae. This integrative approach aims to resolve the systematic uncertainties surrounding this group and provide insights into its evolutionary history.

The Trichoptera fauna of Florida: diversity and distribution

Andrew K. **Rasmussen**[1], Dana R. Denson (retired)[2], Alexander B. Orfinger[3], and Steven C. Harris[4]

1–*Center for Water Resources, Florida A&M University, Tallahassee, Florida, USA* **2–***Reedy Creek Improvement District, Florida, USA* **3–***Department of Life Science, Dalton State College, Dalton, Georgia, USA* **4–***Pennsylvania Western University, Clarion, Pennsylvania, USA*

Corresponding author: Andrew K. Rasmussen (andrew.rasmussen@famu.edu)

The Trichoptera fauna of Florida contains more than 220 species, representing 46 genera within 19 families. The majority of species are widespread in the eastern Nearctic, but a considerable number are endemic to the Southeastern Coastal Plain, with 33 of those species known only from Florida. The most species rich families are the Hydroptilidae (77 species), Leptoceridae (59 species), Hydropsychidae (21 species) and Polycentropodidae (18 species). Species richness is highest in streams of the northern Florida panhandle, which contain a high number of endemic species, disjunct populations of cool-adapted species, as well as wide-ranging species. The water bodies of the Florida peninsula have a less diverse caddisfly fauna but do support many warm-adapted species, a few species of Neotropical origins, and endemic species. Herein we synthesize geographic distributional data of Florida Trichoptera collected from mid-1800s to present by summarizing county-level distribution records for all published specimen data and previously unpublished data. Additionally, we present information on 6 species new to science representing a wide range of genera (*Beraea*, *Ceraclea*, *Hydroptila*, *Oecetis*, *Protoptila*, *Rhyacophila*).

The caddisflies of the Great African Lakes: An example of Lakes Edward and Malawi

Ernesto **Rázuri-Gonzales**

Senckenberg Research Institute and Natural History Museum Frankfurt, Frankfurt am Main, Germany

Corresponding author: Luis Ernesto Rázuri-Gonzales (ernesto.razuri-gonzales@senckenberg.de)

Trichoptera are most diverse in rivers and streams worldwide. Some species also occur along lake shores, with very few occupying the profundal zone. Consequently, most studies focus on running rather than standing waters. The African Great Lakes, located in the East African Rift, are amongst the oldest and largest lakes worldwide and include a staggering diversity of cichlid fishes, one of the most well-known and successful vertebrate radiations. Here, I present preliminary data on the Trichoptera diversity from two of these lakes: Lake

Albert (Uganda) and Lake Malawi (Malawi). Samples were taken with UV light pan traps for 1-2 nights at one location per lake. Eleven genera in five families are recorded at Lake Albert: *Dipseudopsis* (Dipseudopsidae), *Ecnomus* (Ecnomidae), *Aethaloptera*, *Cheumatopsyche* (Hydropsychidae), *Orthotrichia* (Hydroptilidae), *Athripsodes*, *Ceraclea*, *Hemileptocerus*, *Leptocerus*, *Oecetis*, and *Triaenodes* (Leptoceridae). Additionally, the following species were recorded for the first time: *Cheumatopsyche lesnei*, *Aethaloptera dispar* (Hydropsychidae), *Ceraclea corbetti*, *Leptocerus intricatus*, *Oecetis maculata*, and *Triaenodes serratus* (Leptoceridae). Conversely, nine genera in four families were recorded in Lake Malawi: *Dipseudopsis* (Dipseudopsidae), *Ecnomus* (Ecnomidae), *Amphipsyche*, *Cheumatopsyche* (Hydropsychidae), *Hydroptilia*, *Orthotrichia* (Hydroptilidae) *Ceraclea*, *Oecetis*, and *Trichosetodes* (Leptoceridae). The *Hydroptila* specimens from Lake Malawi showed unique morphological characteristics, suggesting a putative new species.

New *Atopsyche* (Hydrobiosidae) from Peru

Ernesto **Rázuri-Gonzales**[1], Ralph W. Holzenthal[2]

1–*Senckenberg Research Institute and Natural History Museum Frankfurt, Frankfurt am Main, Germany*
2–*Department of Entomology, University of Minnesota, St. Paul, Minnesota, USA*

Corresponding author: Luis Ernesto Rázuri-Gonzales (ernesto.razuri-gonzales@senckenberg.de)

Atopsyche, with 146 extant species, is the largest Hydrobiosidae genus. It occurs in the Brazilian Subregion of the Neotropics, Central America, the Antilles, and the southwestern USA. The genus is notably absent from the Chilean Subregion, where a suite of endemic genera replaces it. Currently, 12 species of *Atopsyche* are known from Peru, six of which only occur in this country. Most of these species occur on the eastern flank of the central and southeastern Andes. Over the years, we collected seven additional new species: two species belong to the *falina* and *batesi* species groups in the subgenus *Atopsaura*, two species to the subgenus *Dolochorema*, two species to the *bicolorata* species group (unplaced to subgenus), and one is most similar to *A. cajas* (Schmid considered it as an isolated species). Herein, we present them and discuss their affinities with already described species.

The Trichoptera of Africa: New species, unknowns, and state-of-the-art

Ernesto **Rázuri-Gonzales**[1], Roger J. Blahnik[2], François Ngera Mwangi[3], Steffen U. Pauls[1]

1–*Senckenberg Research Institute and Natural History Museum Frankfurt, Frankfurt am Main, Germany*
2–*Department of Entomology, University of Minnesota, St. Paul, Minnesota, USA* **3**–*Centre des Recherche en Sciences Naturelles Lwiro, Bukavu, Democratic Republic of the Congo*

Corresponding author: Ernesto Rázuri-Gonzales (ernesto.razuri-gonzales@senckenberg.de)

With over 1,300 described species, the Trichoptera of Africa is still one of the most poorly studied faunas worldwide. This project aims to use novel approaches to characterize and accelerate the description of potentially new taxa in the region (Ethiopia, DR Congo, and Togo). One of these approaches is dual-index Illumina sequencing to obtain shorter, 200 bp "mini-barcodes" and the 658 bp fragment obtained by Sanger sequencing. We built a cladogram using 2,140 COI sequences (BOLD data, Sanger COI fragments, and "mini-barcodes"). The data clustered in 341 MOTUs, 215 of which included the newly collected material. We then examined the species diversity in the families Hydropsychidae, Leptoceridae, and Philopotamidae. We identified 43 clusters as potentially new species. Here, we showcase the taxonomic results for the genus *Cheumatopsyche* (Hydropsychidae) and selected genera in the Leptoceridae and Philopotamidae. We recognized some taxonomic issues that need further studies, including possible synonymies and morphological variation in widespread species. Next, holotypes need to be re-examined to solve these problems confidently. The dual-index approach was promising to cluster similar species. However, the use of databases such as BOLD is still difficult since African caddisflies are not well represented.

Elevational biodiversity gradients in caddisflies from the tropical Andes

Blanca **Ríos-Touma**[1], Ralph W. Holzenthal[2], Paul Frandsen[3], Steffen Pauls[4,5,6]

1–*Grupo de Investigación en Biodiversidad, Medio Ambiente y Salud (BIOMAS); Facultad de Ingenierías y Ciencias Aplicadas. Universidad de Las Américas, Ecuador* **2**–*Department of Entomology, University of Minnesota, St. Paul, Minnesota, USA* **3**–*Department of Plant and Wildlife Sciences, Brigham Young University, Provo, Utah, USA* **4**–*Senckenberg Research Institute and Natural History Museum Frankfurt, Frankfurt am Main, Germany* **5**–*Institute of Insect Biotechnology, Justus-Liebig-University, Gießen, Germany* **6**–*LOEWE Centre for Translational Biodiversity Genomics, Frankfurt am Main, Germany*

Corresponding author: Blanca Ríos-Touma (blanca.rios@udla.edu.ec)

The tropical Andes harbor an amazing biological diversity where richness and endemism change across the elevation gradient and the Pacific and Amazonian slopes. Recent evidence from one elevational transect on the Pacific slope of the Ecuadorian Andes suggests that α-diversity of caddisfly species and genera decreases with elevation, while β-diversity increases. To test if this pattern is maintained across the Pacific/Amazon slopes, we assessed caddisfly diversity patterns from four elevation transects (2 on the Pacific slope, 2 on the Amazon slope) at four elevation ranges: 500-700; 1100-1400; 1800-2200; >2800 m asl. We recorded 151 species and 36 genera in total. We found that generic and species composition was mainly driven by elevation, independent of slope: richness of genera and species, as well as abundance, decreased at higher elevations; high altitude communities had a distinct generic diversity when compared with low elevation communities and also between different transects; there was an overlap in species-level composition between the Pacific and the Amazon slopes in lowland sites, and also for middle elevation sites, but only for our southernmost transects, where the cordillera has lower elevations. We detected higher α-diversity at low and middle-low elevations independent of Pacific or Amazonian drainage.

Caddisflies in hot water: a review of climate change studies related to Trichoptera

Desiree Robertson-Thompson

Field Museum of Natural History, Chicago, Illinois, USA

Corresponding author: Desiree Robertson-Thompson (desi.robertson@gmail.com)

Given their critical association with freshwater ecosystems, Trichoptera are thought to be especially vulnerable to climate change. Through a comprehensive literature review, I examine the current landscape of research related to climate change and Trichoptera. The analysis reveals a variety of study types, from field observations, laboratory experiments, and modeling approaches. While many studies speculate on the sensitivity of caddisflies in general to climate change, few focus exclusively on the relationship. Many studies highlight observed changes in macroinvertebrate communities and subsequent ecosystem functioning in response to unusual weather events or habitat alterations over time. Some identify specific climate related threats to caddisflies, such as habitat loss from shrinking glaciers, altered hydrological patterns, or water temperature increases. Other studies explore potential genetic and evolutionary implications of climate change on caddisflies. Despite this breadth, notable gaps remain, particularly in studies focusing on climate adaptation strategies and projecting future range shifts. This presentation serves as both a call to action and an invitation to collaborate, emphasizing the urgent need for targeted research to inform conservation efforts in the face of climate change's impact on Trichoptera.

Assessment of macroinvertebrate communities and water quality in Northwest Quito Metropolitan District

Naomi **Santana**, Carla Díaz, and Rebeca Paredes

Universidad Tecnológica Indoamerica, Quito, Ecuador

Corresponding author: Naomi Santana (tamisantana2002@gmail.com)

Macroinvertebrates serve as pivotal indicators of water quality due to their sensitivity to environmental shifts and ubiquitous presence in aquatic ecosystems. They mirror the conditions of their habitat and water physicochemical properties, offering a reliable assessment tool. In a study within Quito's Metropolitan District, researchers examined three rivers: Pahuma, Pichán, and Mashpi Chico, employing Surber and kick nets for collection. They conducted physical-chemical water analyses and habitat assessments alongside macroinvertebrate identification. The study utilized diverse indices like QBR-And, IHF, Shannon, BMWP/Col, and EPT to evaluate water quality and macroinvertebrate diversity. Results revealed superior water quality in Mashpi and Pahuma, marked by higher species diversity and better water quality indices compared to Pichán. The presence of pollution-sensitive macroinvertebrates indicated healthier aquatic habitats in Mashpi and Pahuma. Despite data processing challenges, discernible disparities in water quality among the rivers were evident, suggesting human interference in Pichán contributing to its inferior quality. The study underscores the necessity of conserving natural areas and implementing monitoring and mitigation strategies to safeguard aquatic ecosystems' health and biodiversity.

On the Andean endemic genus *Scotiotrichia* Mosely (Trichoptera: Glossosomatidae: Protoptilinae): description of the larva and biogeographic patterns of the Andean Protoptilinae

Julieta V. **Sganga**[1], Cecilia Brand[2,3], Juan J. Morrone[4], Danielle Anjos-Santos[2,3]

1–*Departamento de Biodiversidad y Biología Experimental, Facultad de Ciencias Exactas y Naturales, Universidad de Buenos Aires, Buenos Aires, Argentina* **2**–*Centro de Investigaciones Esquel de Montaña y Estepa Patagónica (CIEMEP-CONICET-UNPSJB), Esquel, Argentina* **3**–*Facultad de Ciencias Naturales y Ciencias de la Salud, Universidad Nacional de la Patagonia "San Juan Bosco", Esquel, Argentina* **4**–*Museo de Zoología 'Alfonso L. Herrera', Facultad de Ciencias, Universidad Nacional Autónoma de México (UNAM), Mexico City, Mexico*

Corresponding author: Julieta V. Sganga (jsganga@gmail.com)

Scotiotrichia Mosely is a monospecific genus of the subfamily Protoptilinae, endemic to the Andean region. *Scotiotrichia ocreata* is known exclusively from the adults and has been recorded from the Patagonia of Argentina and Chile. To associate the larva of this species, we examined adults collected in the Chubut province using Malaise and light traps and larvae and metamorphotypes collected manually or with a Surber net sampler. The larva of *Scotiotrichia ocreata* is characterized by the morphology of the tarsal claws, with basal process short and thick, arising perpendicular to the base of the claw, and basal seta long and broad; the chaetotaxy of the legs, especially the number and distribution of feathered setae; the presence of three accessory teeth on the anal prolegs; and six pairs of long setae on abdominal tergum IX. Additionally, we gathered information regarding the distribution of all known Protoptilinae species found in the Andean region *sensu lato* to study their distributional patterns using a track analysis. We found two generalized tracks, one austral, corresponding to the Subantarctic subregion, and another northern, at the Páramo province of the South American transition zone. These tracks represent ancestral biotas that were fragmented by geologic or climatic events.

Exploring the diversity of caddisflies (Insecta: Trichoptera) in Patagonia: new records and taxonomic insights from Argentina

Julieta V. **Sganga**[1], Gleison R. Desidério[2], Camila Fornis[1], María Laura Libonatti[1], Fabián G. Jara[3], Gabrielle Jorge[4], and Neusa Hamada[4]

1–*Universidad de Buenos Aires, Facultad de Ciencias Exactas y Naturales, Departamento de Biodiversidad y Biología Experimental, Buenos Aires, Argentina* **2**–*Instituto Nacional de Pesquisas da Amazônia (INPA), Programa de Apoio à Fixação de Jovens Doutores no Brasil (PROFIX-JD), Lab de Citotaxonomia e Insetos Aquáticos (LACIA), Manaus, Brazil* **3**–*Grupo de Ecología de Macroinvertebrados Acuáticos, Instituto de Investigaciones en Biodiversidad y Medioambiente INIBIOMA (UNComahue-CONICET), San Carlos de Bariloche, Río Negro, Argentina* **4**–*Instituto Nacional de Pesquisas da Amazônia (INPA), Coordenação de Pós-Graduação (COPOG), Programa de Pós-Graduação em Entomologia (PPGEnto), Lab de Citotaxonomia e Insetos Aquáticos (LACIA), Manaus, Brazil;*

Corresponding author: Julieta V. Sganga (jsganga@gmail.com)

The Argentinian Patagonia stands out as a significant area for biodiversity studies due to its high degree of endemic species and its zoogeographical connections with other austral areas worldwide, instead of the Neotropical region that extends in the rest of the country. Our study focused on samples collected from Río Negro and Chubut provinces, using Malaise and light traps near streams and wetlands, as well as D-net collections in wetlands near Bariloche City. We identified 22 caddisfly species grouped in 15 genera and 10 families. Notably, *Sortosa bispinosa* (Flint) (Philopotamidae) and *Notidobiella inermis* Flint (Sericostomatidae), previously known only from Chile, are recorded herein for the first time from Argentina, with the latter marking the first record of the genus *Notidobiella* in the country. Furthermore, we observed intraspecific variation in the male genitalia of *Brachysetodes quadrifidus* Schmid (Leptoceridae), specifically in the shape of the parameres, and provided a detailed redescription of the male of *Verger michaelseni* (Ulmer) (Limnephilidae), along with the first description of its female identified through larval association. These findings expand the known caddisfly fauna of Argentina to 335 species, 81 genera and 18 families, with Patagonia alone contributing 67 species in 32 genera and 13 families.

Waterfalls as a reservoir for caddisfly larvae: exploring a poorly known habitat

Darha **Solano-Ulate**[1], Monika Springer[2,3]

1–*Escuela de Biología y Centro de Investigación en Contaminación Ambiental (CICA), Universidad de Costa Rica, San José, Costa Rica* **2**–*Escuela de Biología, Centro de Investigación en Biodiversidad y Ecología Tropical (CIBET), Universidad de Costa Rica, San José, Costa Rica* **3**–*Escuela de Biología, Centro de Investigación en Ciencias del Mar y Limnología (CIMAR), Universidad Costa Rica, San José, Costa Rica*

Corresponding author: Darha Solano-Ulate (darha.solano@ucr.ac.cr)

Waterfalls, due to certain physical characteristics, have not been thoroughly studied as a habitat for freshwater macroinvertebrates, and in some cases, they appear to be an exclusive environment for certain species. In order to better understand the role of waterfalls as an aquatic habitat in Costa Rica, macroinvertebrates were collected in 38 waterfalls across the country through direct collecting and rappel techniques. Caddisfly larvae represented a third of the total collected, with 10,602 individuals from 10 families. *Calosopsyche* (Hydropsychidae) was one of the most abundant and frequent genera, suggesting a strong preference for this habitat. Larvae from *Atanatolica* (Leptoceridae) and Xiphocentronidae were quite common on hygropetric zones, while *Wormaldia* (Philopotamidae), *Contulma* (Anomalopsychidae), *Cerasmatrichia* and *Alisotrichia* (Hydroptilidae), despite uncommon in rivers throughout the country, were found abundantly on waterfalls. Larvae of *Chimarra* were also found, building similar shelters to *Wormaldia*. Furthermore, a peculiar larval morphotype from *Helicopsyche* was found living in hygropetric zones of waterfalls, which had not been observed before in rivers and streams. The results of this study represent an important baseline for defining new collection sites of adult caddisflies, and for the association and description of their larvae stages, possibly unknown due to this understudied habitat.

Integrating genetic diversity in temporal insect monitoring: The example of three freshwater invertebrate species in the Bavarian Forest National Park

Jan Simon Stark, Oskar Schröder, Steffen U. **Pauls**

Senckenberg Research Institute and Natural History Museum Frankfurt, Frankfurt am Main, Germany

Corresponding author: Steffen U. Pauls (steffen.pauls@senckenberg.de)

Temporal genomic studies, which utilize samples from various time points, provide a unique avenue to directly observe such genetic variations. This study provides an example of incorporating intraspecific genetic diversity into global change monitoring. We assess the genetic diversity and spatiotemporal population structure of the mayfly *Baetis alpinus*, the stonefly *Brachyptera seticornis*, and the caddisfly *Drusus discolor* within three distinct river systems in the Bavarian Forest National Park (BFNP) from 2016 through 2023. Nuclear microsatellite analysis was employed for nine loci for each organism. A total of 735 *B. alpinus*, 295 *B. seticornis*, and 193 *D. discolor* individuals were genotyped to assess local population structures and identify any temporal changes. The results revealed distinct spatial genetic patterns in *B. alpinus*. In contrast, *B. seticornis* and *D. discolor* lacked significant spatial structure, indicating maintained gene flow throughout the BFNP. Despite the observed differences in spatial patterns, all three species exhibited stability in their genetic structures throughout the study period. The data underscores the importance of species-specific approaches when investigating community structures and highlights the need for extended temporal genomic analyses, particularly considering ongoing global change dynamics affecting biodiversity.

Trichoptera and Citizen Science in the Netherlands

David **Tempelman**, María J. Sanabria

Semblis Foundation, JL Vught, The Netherlands

Corresponding author: David Tempelman (davidtempelman67@gmail.com)

In 2023, over 30,000 records of Trichoptera were submitted to the citizen science portal Observation.org in the Netherlands. A large proportion of the records concern species with a summer diapause as imago. When compared with data from previous years, the number of these species seems to be on the rise. On the other hand, numerous species without diapause did not see such an increase over the last few years. To gain more insight in this matter, an analysis was made of Trichoptera data submitted to Observation.org over 2005-2023. In 2005, species without a diapause where the dominant group, making up circa 80% of the total Trichoptera population, while species with a diapause amounted to 20%. From 2005 onwards, the proportion of species with a diapause gradually increased, and in 2023, both groups are nearly equal in proportion. It is concluded that data, gathered from the citizen science platform are well suited for monitoring changes in Trichoptera species composition. It is likely that analyses of these data also may give more insight in understanding relations between Trichoptera species and their numbers and climatic changes.

Microcaddisflies, morphology, and modern molecular methods: collections-based research to establish the microcaddisfly phylogeny

Robin Thomson

Department of Entomology, University of Minnesota, St. Paul, Minnesota, USA

Corresponding author: Robin Thomson (thom1514@umn.edu)

The family Hydroptilidae is an extremely diverse family within Trichoptera that displays a wide array of ecological, morphological, and habitat diversity. In terms of species diversity, Hydroptilidae is the largest family in the order, containing over 2,600 known species found in all faunal regions of the world. However, little is known about microcaddisfly evolutionary history that is supported by current phylogenetic methods. A stable phylogenetic framework based on statistically supported methods is needed to consistently define taxa and provide context for how they relate to each other and are arranged within the family overall. A total-evidence (molecular + morphology) phylogeny of the family Hydroptilidae will be constructed, including the first morphological assessment of homology across the family and the first molecular dataset to cover the entire family using targeted enrichment techniques, to evaluate the monophyly of the traditionally recognized subfamilies within Hydroptilidae and to infer the evolutionary relationships of the included taxa. In addition to the ability to place and describe new biodiversity, a stable classification will make it possible to pursue numerous research directions, such as character evolution within Hydroptilidae, evolutionary trends across Trichoptera, and the opportunity to integrate sequence data with collections-based data.

Sublethal effects of two heavy metals on *Nectopsyche* sp. (Trichoptera: Leptoceridae) in Andean rivers

Christian **Villamarín**[1], Agnes Lohs[2], Mishell Donoso[1], Blanca Ríos-Touma[1], Pablo Castillejo[1], Melanie Loachamin[1], Milton Sosa[1]

1–*Grupo de Investigación Biodiversidad, Medio Ambiente y Salud (BIOMAS), Facultad de Ingenierías y Ciencias Aplicadas, Ingeniería Ambiental, Universidad de Las Américas, Quito, Ecuador* **2**–*Toxicology Master Program, Duisburg Essen University, Germany*

Corresponding author: Christian Villamarín (christian.villamarin@udla.edu.ec)

In Ecuador, mining activities have expanded due to increased mining concessions and illegal mining and the sublethal effects of heavy metals on local aquatic biota is little known. Our study conducted microcosm experiments, evaluating the sublethal effects on *Nectopsyche* sp. (Leptoceridae) through behavioral measurements and biomarkers as an effect of different concentrations of Mercury (Hg) and Arsenic (As). The experiment evaluated concentrations of different regulation values for Hg and As and assessed the effect on individuals for seven days under controlled conditions. Laboratory experiments evaluated the mortality and mobility of the individuals and measured biomarkers such as Catalase activity, Glutathione S-Transferase activity, and Ferric Reducing Antioxidant Power. Mortality did not show significant differences. However, mobility showed significant differences at the highest concentrations. Chronic exposure to Hg increased CAT, GST, and FRAP, but only GST exhibited significant differences. For its part, exposure to As caused a decrease in all measured biomarkers, and CAT and FRAP showed significant differences at the highest concentrations. The results suggest that more detailed studies should be carried out using biomarkers to define the actual effects on freshwater biodiversity caused by mining and define MPLs that do not negatively affect populations in the long term.

Spatial distribution of Trichoptera larvae in the Cajas Massif lakes

Diego **Vimos**[1], Pablo V. Mosquera[2,3], Henrietta Hampel[1], and Raúl F. Vazquez[4]

1–*Laboratorio de Ecología Acuática, Facultad de Ciencias Químicas, Universidad de Cuenca, Cuenca, Ecuador* **2**–*Departament de Biologia Evolutiva, Ecologia i Ciències Ambientals, Universitat de Barcelona, Barcelona, Spain* **3**–*Subgerencia de Gestión Ambiental de la Empresa Pública Municipal de Telecomunicaciones, Agua Potable, Alcantarillado y Saneamiento (ETAPA EP), Cuenca, Ecuador* **4**–*Departamento de Ingeniería Civil, Facultad de Ingeniería, Universidad de Cuenca, Cuenca, Ecuador*

Corresponding author: Diego Vimos (diego.vimos@ucuenca.edu.ec)

A significant amount of studies on the diversity and ecology of Trichoptera communities has been performed in streams and rivers; nevertheless, very little is known on Trichoptera communities in tropical high mountain lentic water bodies. Therefore, this research aimed at assessing the main variables that restrict the spatial distribution of Trichoptera communities present in the littoral areas of lakes in the Cajas Massif, southern Ecuador. Twenty-four environmental variables (hydro-morphological, water quality and microhabitat) were monitored and macroinvertebrate samples were collected using a hand net in 202 study lakes. The order Trichoptera was present in 65.5% of the lakes and represented 1.6% of the total abundance of the benthic community. *Oxyethira* was the most representative genus, followed by *Metrichia* and *Anomalocosmoecus*. The application of the Partial Canonical Correspondence Analysis and the Generalized Additive Model revealed that altitude was the most influential hydro-morphological variable in the distribution of the *Oxyethira*, *Anomalocosmoecus*, *Metrichia* and *Nectopsyche*. Influential water quality variables were ammonium (*Oxyethira*, *Helicopsyche*, *Metrichia* and *Anomalocosmoecus*) and dissolved oxygen (*Metrichia* and *Anomalocosmoecus*). Furthermore, microhabitat scale variables did not have any influence on the Trichoptera communities. Therefore, given the ecological role that these insects play and their sensitivity to changes in their habitat, the distribution and diversity patterns observed in this research are likely to contribute to a better ecological knowledge of these very important tropical high mountain water bodies, which could finally contribute to a better conservation of these important aquatic ecosystems of the Ecuadorian Andes.

Estimate of the life history and the net spinning habit on *Macrostemum radiatum* (McLachlan 1872) in the central Honshu Island of Japan

Shozo Watanabe

6-5, 4chome, Karatodai, Kitaku, Kobe, Hyogo Prefecture, Japan

Corresponding author: Shozo Watanabe (shozowatanabe@gmail.com)

The life history and the habit of the net-spinning caddisfly, *Macrostemum radiatum* (McLachlan 1872), was studied in a midstream of the Kakogawa River, central Honshu Island of Japan, 2019-2022. This population seems to be bivoltine, or it may be trivoltine through five larval instars with an adult period from spring to autumn. The density and abundance of this species varied seasonally from 64 to 368 individuals/m^2, from 0.65 to 18.4 g/m^2, respectively, both of which were minimal in summer. However, those were not significantly different between the center and the river's shore. The body in wet weight of the larvae increased proportionally with the logarithm of body size of the larvae: ww=4.34 hw 3.69 (herein ww: wet weight, hw: head capsule width). Those of the final instar larvae decreased from spring to summer and subsequently increased from autumn to winter. The larvae examined in the laboratory constructed their retreat using silk with or without sand grains and also constructed capture nets using silk under stirring conditions.

18th International Symposium on Trichoptera

Quito, Ecuador 1-5.vii.2024

Support provided by:

World Wildlife Fund, Ecuador
Nature Experience
Semblis Foundation

WWF

nature experience
nature and scientific tour

Semblis foundation
